# Three-Dimensional Architecture and Biogenesis of Membrane Structures Associated with Hepatitis C Virus Replication

**DOI:** 10.1371/journal.ppat.1003056

**Published:** 2012-12-06

**Authors:** Inés Romero-Brey, Andreas Merz, Abhilash Chiramel, Ji-Young Lee, Petr Chlanda, Uta Haselman, Rachel Santarella-Mellwig, Anja Habermann, Simone Hoppe, Stephanie Kallis, Paul Walther, Claude Antony, Jacomine Krijnse-Locker, Ralf Bartenschlager

**Affiliations:** 1 Department of Infectious Diseases, Molecular Virology, University of Heidelberg, Heidelberg, Germany; 2 European Molecular Biology Laboratory, Heidelberg, Germany; 3 Electron Microscopy Core Facility, University of Heidelberg, Heidelberg, Germany; 4 Central Electron Microscopy Facility, Ulm University, Ulm, Germany; University of Alabama at Birmingham, United States of America

## Abstract

All positive strand RNA viruses are known to replicate their genomes in close association with intracellular membranes. In case of the hepatitis C virus (HCV), a member of the family *Flaviviridae*, infected cells contain accumulations of vesicles forming a membranous web (MW) that is thought to be the site of viral RNA replication. However, little is known about the biogenesis and three-dimensional structure of the MW. In this study we used a combination of immunofluorescence- and electron microscopy (EM)-based methods to analyze the membranous structures induced by HCV in infected cells. We found that the MW is derived primarily from the endoplasmic reticulum (ER) and contains markers of rough ER as well as markers of early and late endosomes, COP vesicles, mitochondria and lipid droplets (LDs). The main constituents of the MW are single and double membrane vesicles (DMVs). The latter predominate and the kinetic of their appearance correlates with kinetics of viral RNA replication. DMVs are induced primarily by NS5A whereas NS4B induces single membrane vesicles arguing that MW formation requires the concerted action of several HCV replicase proteins. Three-dimensional reconstructions identify DMVs as protrusions from the ER membrane into the cytosol, frequently connected to the ER membrane via a neck-like structure. In addition, late in infection multi-membrane vesicles become evident, presumably as a result of a stress-induced reaction. Thus, the morphology of the membranous rearrangements induced in HCV-infected cells resemble those of the unrelated picorna-, corona- and arteriviruses, but are clearly distinct from those of the closely related flaviviruses. These results reveal unexpected similarities between HCV and distantly related positive-strand RNA viruses presumably reflecting similarities in cellular pathways exploited by these viruses to establish their membranous replication factories.

## Author Summary

All positive-strand RNA viruses replicate in the cytoplasm in distinct membranous compartments acting as ‘replication factories’. Membranes building up these factories are recruited from different sources and serve as platforms for the assembly of multi-subunit protein complexes (the ‘replicase’) that catalyze the amplification of the viral RNA genome. In this study we found that hepatitis C virus (HCV), a major causative agent of chronic liver disease, induces profound remodeling of primarily endoplasmic reticulum-derived membranes. Surprisingly, the 3D architecture of these membrane rearrangements is similar to those induced by the unrelated picorna- and coronaviruses, but in striking contrast to the closely related flaviviruses. Early in infection HCV induces double membrane vesicles (DMVs) that emerge as protrusions of the ER; later on, HCV induces in addition multi-membrane vesicles that are probably the result of a cellular stress reaction and that are reminiscent to an autophagic response. These profound membrane rearrangements are induced by the concerted action of HCV-encoded nonstructural proteins of which NS5A is the only one capable to induce DMVs. These results provide important insights into the 3D architecture of the membrane alterations induced by HCV and reveal unexpected similarities between HCV and the very distantly related picorna- and coronaviruses.

## Introduction

Hepatitis C virus (HCV) infection affects ∼170 million people worldwide and is a major cause of chronic liver disease including liver cirrhosis and hepatocellular carcinoma [1], [2]. In spite of the recent approval of the first generation of HCV-specific protease inhibitors, current therapeutic options are still limited by profound side effects and eventually antiviral drug resistance [3]. Thus, there is an urgent need to develop novel selective antiviral strategies, for which fundamental understanding of the basic principles of HCV replication is essential.

HCV is a positive-strand RNA virus belonging to the family *Flaviviridae* (genus *Hepacivirus*). The viral genome, ∼9.6 kb in length, is an uncapped linear molecule that contains a single open reading frame (ORF) flanked by 5′ and 3′ non-translated regions (NTRs) [4]. After release of the viral RNA genome into the cytoplasm, it serves as messenger RNA and is used for cap-independent translation *via* the internal ribosome entry site (IRES) located within the 5′NTR (reviewed in [5]). The resulting polyprotein is co- and post-translationally processed by cellular and viral proteases into 10 different proteins that are required for RNA replication and virion formation. The N-terminal region of the polyprotein comprises the structural proteins core as well as envelope proteins 1 and 2 (E1 and E2) that build up the virus particle. C-terminal of E2 are p7 and nonstructural protein 2 (NS2) that are required for assembly of infectious HCV particles (reviewed in [6]). The latter is in addition a cysteine protease responsible for cleavage between NS2 and NS3 (reviewed in [4]). The minimal HCV replicase comprises the remaining nonstructural proteins: NS3, NS4A, NS4B, NS5A and NS5B [7]. In fact, subgenomic RNAs (replicons) composed of only the NTRs and the region encoding for these replicase proteins are capable of autonomous replication in the human hepatoma cell line Huh7.

A common feature of all positive-strand RNA viruses is the remodeling of intracellular membranes creating mini-organelles or ‘replication factories’ where RNA amplification and eventually also virion assembly take place (reviewed in [8]). Formation of such sites facilitates coordination of the different steps of the replication cycle, but might also shield viral RNA, especially double strand (ds) RNA replication intermediates, from recognition by innate sensors such as RIG-I (retinoic acid-inducible gene I, also known as DDX58) or MDA5 (melanoma differentiation-associated gene 5, also known as IFIH1 or Helicard). In the case of flaviviruses such as Dengue virus or West Nile virus, it has been shown that RNA replication occurs most likely within membrane invaginations originating from the endoplasmic reticulum (ER) [9], [10]. Similar invaginations have been described e.g. for Flock House virus or Semliki Forest virus, although in these cases membrane alterations occur at other sites: the outer mitochondrial membrane or the plasma membrane, respectively [11], [12]. In contrast, in case of the poliovirus, the prototype member of the picornaviruses, complex membrane rearrangements have been described that are formed most likely as protrusions originating from *cis*-Golgi membranes and transforming in a time-dependent manner from single membrane tubular compartments into double-membrane structures [13]. Likewise, coronaviruses [14], [15] and arteriviruses [16], [17] induce double membrane vesicles (DMVs) that resemble exvaginations of ER-derived membranes.

In case of HCV, membrane rearrangements with a ‘membranous web’ (MW)-like appearance [18], [19] were originally detected in cells over-expressing the viral polyprotein, or only NS4B. Morphologically the MW is a cytoplasmic accumulation of highly heterogeneous membranous vesicles that are embedded into an amorphous matrix. A recent study suggests that the predominant structures within the MW are DMVs and less frequently, multivesicular membranes [20]. However, the 3D architecture of the MW and its biogenesis are not known and it is unclear where precisely viral RNA replication occurs.

Taking advantage of a combination of confocal microscopy, electron microscopy (EM) and electron tomography (ET), in this study we have dissected the composition, 3D architecture and biogenesis of the various HCV-induced membrane alterations. The results suggest that HCV and the distantly related picorna- and coronaviruses, but not the closely related flaviviruses, share strikingly similar morphology of remodeled intracellular membranes likely reflecting the exploitation of common host cell pathways by these viruses.

## Results

### HCV proteins localize to multiple membranous cell compartments

**Figure 1:**
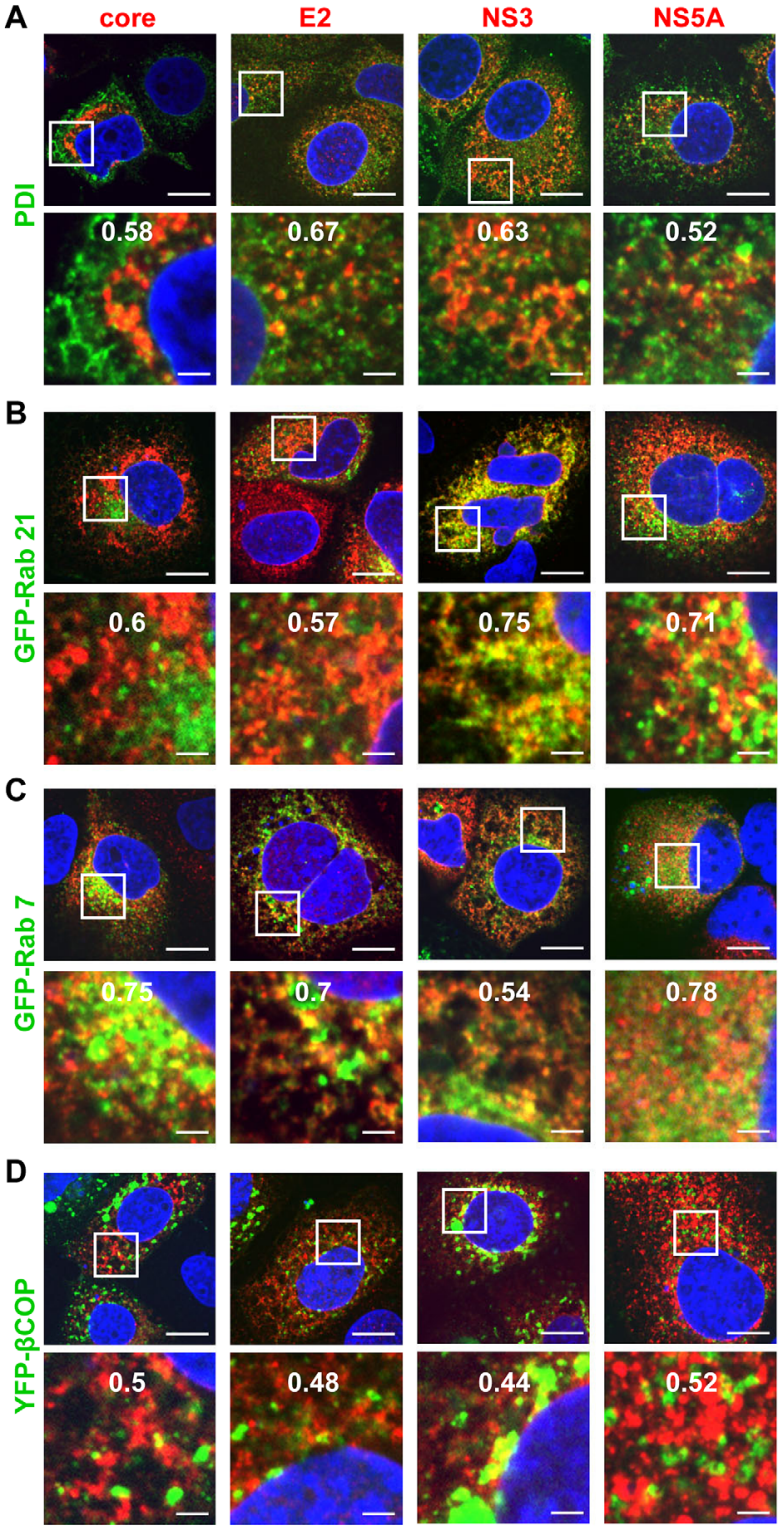
Colocalization of HCV Proteins with organelle-specific markers in HCV infected cells. Huh7 cells were infected with HCV (strain Jc1) using 30 TCID_50_/cell and 48 h later cells were fixed and processed for fluorescence microscopy. In case of samples shown in panels B–D, cells were first transfected with expression constructs specified in the left of each panel and 24 h later cells were infected as described above. Samples were analyzed with a Nikon TE2000-E inverted confocal microscope at 60× magnification. (A)–(D) Colocalization of HCV proteins specified in the top of each panel with protein disulphide isomerase (PDI; an ER marker), GFP-Rab21 (marker for early endosomes), GFP-Rab7 (marker for late endosomes) or βCOP-YFP (marker for COP I vesicles). The upper panels represent a low magnification overview; boxed areas are shown as enlargement in the corresponding panel below. The nucleus was stained with DAPI (blue). Scale bars represent 10 µm (top panels) and 2 µm (lower panels). The quantification of the degree of colocalization (Pearson's correlation coefficient) is given at the top of the enlarged pictures.

To gain insight into the origin of the MW and its composition with respect to involved subcellular compartments, we conducted an extensive series of colocalization studies by using immunofluorescence (IF) microscopy. HCV proteins were detected in Huh7 cells 48 hours post infection (hpi) by using mono-specific antisera and cellular proteins were detected by antibodies recognizing endogenous proteins or by ectopic expression of GFP-tagged proteins (for details of used antisera and constructs see Table S1 in Text S1). Subcellular compartments tested in this way included ER, lipid droplets (LDs), mitochondria, early and late endosomes, lysosomes, ER-Golgi intermediate compartment and others (for a complete summary of tested markers and their abbreviations see Table 1). Confocal images were analyzed by determining Pearson's correlation coefficient which is a measure for the degree of overlap of the two images recorded in different channels and thus indicative for a biological interaction [21], [22]. Only values higher than 0.5 were considered as colocalization. All examined HCV proteins (core, E2, NS3, NS4B and NS5A, but not NS5B for which no suitable antibody was available) colocalized with PDI, a marker for the rough (r) ER (Figure 1A and Table 1). Similar results were obtained with another rER marker protein: GRP94 (Table 1). HCV proteins also colocalized with Rab21 and Rab7A, which are markers for early and late endosomes, respectively (Figure 1B, C). Moreover, some HCV proteins colocalized with markers for COP I (β-COP) or COP II (sec13) vesicles (Figure 1D and supplementary Figure S1C, respectively). Colocalization was also found between HCV proteins and mitochondria as revealed by staining with MitoTracker Red (Figure S1A) as well as between core, NS4B and NS5A and LDs that were detected by ADRP staining (Figure S1B) or with the lipid dye BODIPY (Table 1). Neither GFP-LC3, a marker for autophagosomes, (Figure S1D), nor any of the markers for Golgi and lysosomes colocalized with viral proteins (Table 1). We note that analogous results were obtained when cells were analyzed already 24 hpi (supplementary Figure S2), although at this time point HCV-specific signals were very much reduced as compared to 48 hpi. In summary, these data suggest that membrane alterations induced by HCV originate primarily from the ER, but contain in addition membranes derived from other subcellular compartments such as early and late endosomes, as well as COP I/II transport vesicles. In addition, the MW contains mitochondrial membranes and LDs, the latter playing an important role in HCV assembly [6].

**Table 1:** Summary of immunofluorescence-based colocalization studies of HCV proteins and cellular marker proteins.

cellular proteins			viral proteins				
organelle		marker	core	E2	NS3	NS4B	NS5A
		dsRNA	0.41	0.3	0.34	0.23	0.36
rough ER		PDI	0.58	0.67	0.63	0.53	0.52
		GRP94	0.72	0.58	0.74	0.54	0.43
smooth ER		EH	0.34	n.a.	0.43	n.a.	0.35
reticular subdomain of rough ER		CLIMP-63	0.37	0.39	0.59	0.323	0.4
LD		BODIPY	0.6	0.36	0.32	0.54	0.37
		ADRP	0.82	0.42	0.42	0.44	0.56
mitochondria		MitoTracker	0.48	0.53	0.6	0.62	0.5
ER-Golgi intermediate compartment		GFP-ERGIC-53	0.3	0.31	0.31	n.a.	0.34
*cis* Golgi network		GOS-28	0.29	0.53	0.34	0.24	0.4
*trans* Golgi network		TGN46	0.27	n.a.	0.23	n.a.	0.24
early endosome		GFP-Rab21	0.6	0.57	0.75	n.a.	0.71
		Rab5	0.27	n.a.	0.26	n.a.	0.30
		EEAI	0.2	n.a.	0.16	n.a.	0.28
autophagosome		GFP-LC3	0.21	0.21	0.14	n.a.	0.03
vesicular trafficking	COP I	YFP-β COP	0.5	0.48	0.44	n.a.	0.52
	COP II	Sec13	0.43	n.a.	0.34	n.a.	0.35
recycling compartment		Rab11	0.3	n.a.	0.39	n.a.	0.36
		GFP-Rab11	0.33	0.63	0.28	n.a.	0.51
late endosomes		Lamp-3	0.32	0.17	0.19	0.19	0.25
		GFP-Rab7A	0.75	0.7	0.54	n.a.	0.78
multivesicular bodies		LBPA	0.40	0.30	0.36	0.3	0.3

Naïve high-passage Huh-7 cells were infected with 30 TCID_50_/cell of Jc1 and 48 h later processed for indirect immunofluorescence. Signal intensities were quantified by using Intensity Correlation Analysis (ICA) of the Image J software package and are given as Pearson's correlation coefficient (PCC). PCC can range from −1 to 1, corresponding to no or perfect colocalization. Only values higher than 0.5 were considered as colocalization.

n.a., not applicable owing to incompatible antibodies.

ADRP, adipose differentiation-related proteins or adipophilin; CLIMP-63, cytoskeleton linking membrane protein 63; COP I, coat protein I; COP II, coat protein II; EEA1, early endosome antigen 1; EH, epoxide hydrolase; ERGIC-53, ER Golgi intermediate compartment; GOS-28, Golgi SNARE protein 28; GRP94, glucose related protein 94; Lamp-3, lysosome-associated membrane protein-3; LBPA, lysobisphosphatidic acid; LC-3, light chain protein 3; PDI, protein disulphide isomerase; Rab5, Ras-related GTP binding protein 5; Rab7A, Ras-related GTP binding protein 7A; Rab11, Ras-related GTP binding protein 11; Rab21, Ras-related GTP binding protein 21;. sec13, secretory protein 13; TGN46, *Trans* Golgi network protein 46.

### Correlative microscopy identifies sites of high fluorescence as complex HCV-induced membranous compartments

**Figure 2:**
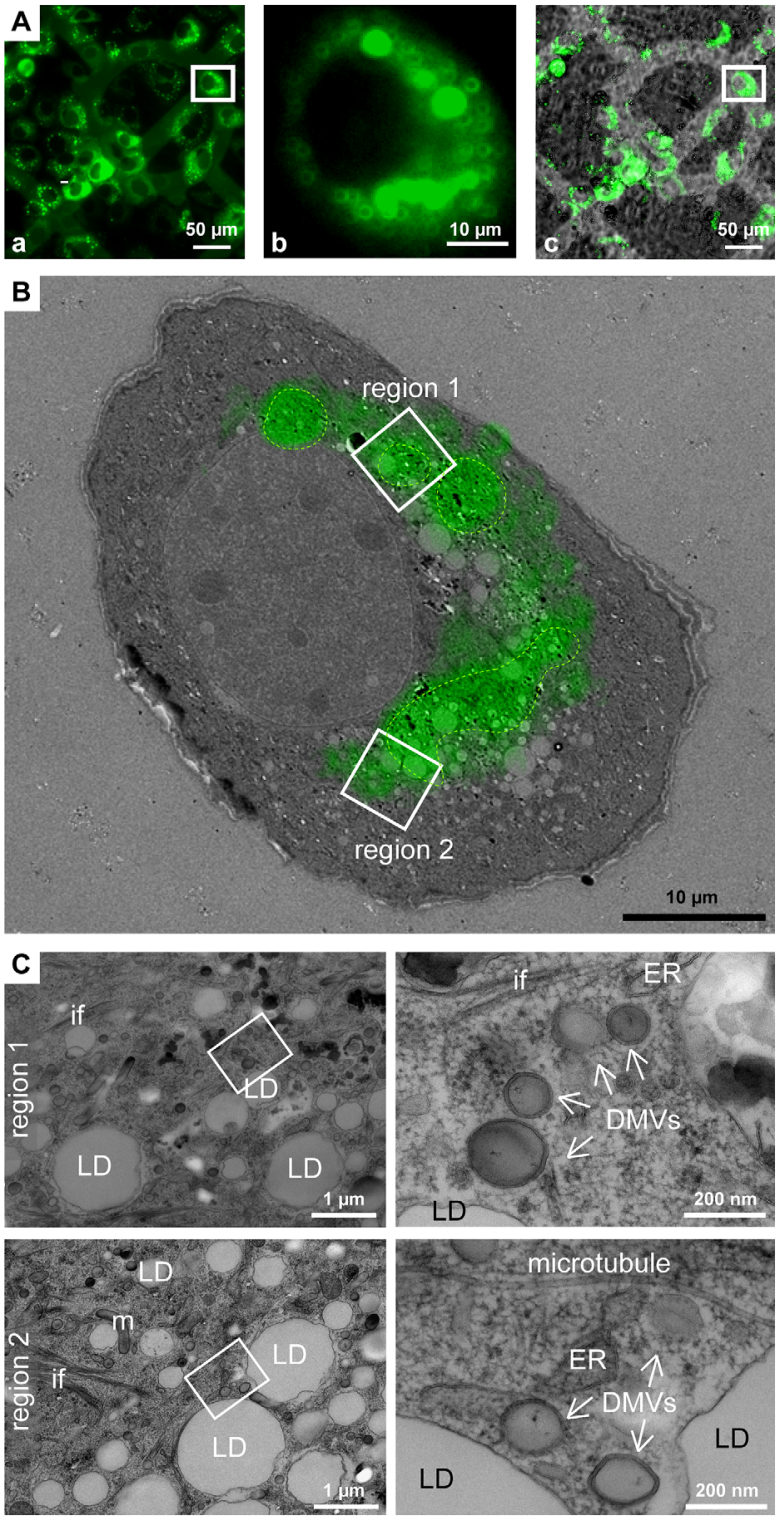
Correlative light-electron microscopy of cells containing a GFP-tagged subgenomic replicon. (A) Epifluorescence microscopy of live cells containing a subgenomic replicon with a GFP-tagged NS5A. Huh7-Lunet cells were transfected with replicon RNA and seeded onto carbon-patterned sapphire discs. Twenty-four hours later cells were analyzed by fluorescence microscopy and immediately processed for EM. (a) Fluorescence image; (b) enlarged fluorescence image of the cell of interest; (c) merge of bright field and fluorescence images. Coordinates etched onto the surface of the sapphire disc were used to record the position of the selected cells. White squares in a and c enclose the cell shown in b. (B) EM micrograph of the cell boxed in panel Ab overlapped with the fluorescence image. Areas marked with a green dotted line indicate regions of intense fluorescence. Note that the images do not match perfectly because the fluorescence image corresponds to the complete cell whereas the EM image represents one 60 nm ultrathin section of the same cell. (C) Higher magnification images of the two different regions, labeled 1 and 2 in panel B, corresponding to regions with intense fluorescence (1) or a region corresponding to the intersection of high to low fluorescence (2). Region 1 (top panel) corresponds to a DMV-containing area residing in close proximity of the ER; region 2 (bottom panel) corresponds to a LD-enriched area containing DMVs in very close proximity. Areas marked with white squares in the left images are magnified in the corresponding right panels. LD, lipid droplet; ER, endoplasmic reticulum; DMV, double membrane vesicle; m, mitochondrium; if, intermediate filaments.

A main limitation of fluorescence microscopy is the difficulty to allocate viral and cellular proteins to distinct subcellular structures. For instance, it is unclear whether HCV-positive punctae, frequently observed in infected or transfected cells represent mere accumulations of viral proteins or distinct membranous compartments. To overcome this limitation we conducted correlative light electron microscopy (CLEM) by using a replication-competent subgenomic replicon containing a GFP-tagged NS5A [23]. Relevant fluorescent structures were allocated by fluorescence microscopy of live cells grown on patterned sapphire discs (Figure 2A) and cells were immediately processed for EM (Figure 2B). We found that highly fluorescent GFP-positive areas often corresponded to accumulations of double membrane vesicles (DMVs) surrounding LDs and residing in close proximity of ER (Figure 2C). These structures were not observed in mock-infected cells, arguing for an HCV-specific phenotype. We therefore concluded that the highly fluorescent structures are not mere aggregates of viral proteins, but instead correspond to complex HCV-induced membranous compartments containing LDs and DMVs.

### HCV infection induces massive rearrangements of intracellular membranes in a time-dependent manner

With the aim to study HCV-induced alterations of intracellular membranes at the ultrastructural level, we first defined the EM method giving best preservation of the MW. We compared embedding of cells grown on glass coverslips or prepared as a pellet and embedded in epon (Protocol S1 in Text S1) with embedding of cells grown on sapphire discs and preserved by high pressure freezing – freeze substitution (HPF-FS) prior to embedding into epon (supplementary Figure S3). Best preservation of membranes was achieved with chemical fixation of cells (which was required for biosafety reasons to inactivate infectious HCV), prior to HPF-FS and subsequent embedding of cells into epon resin (supplementary Figure S3C). Under these conditions membrane bilayers were clearly discernable and therefore, this method was used for all EM analyses.

**Figure 3:**
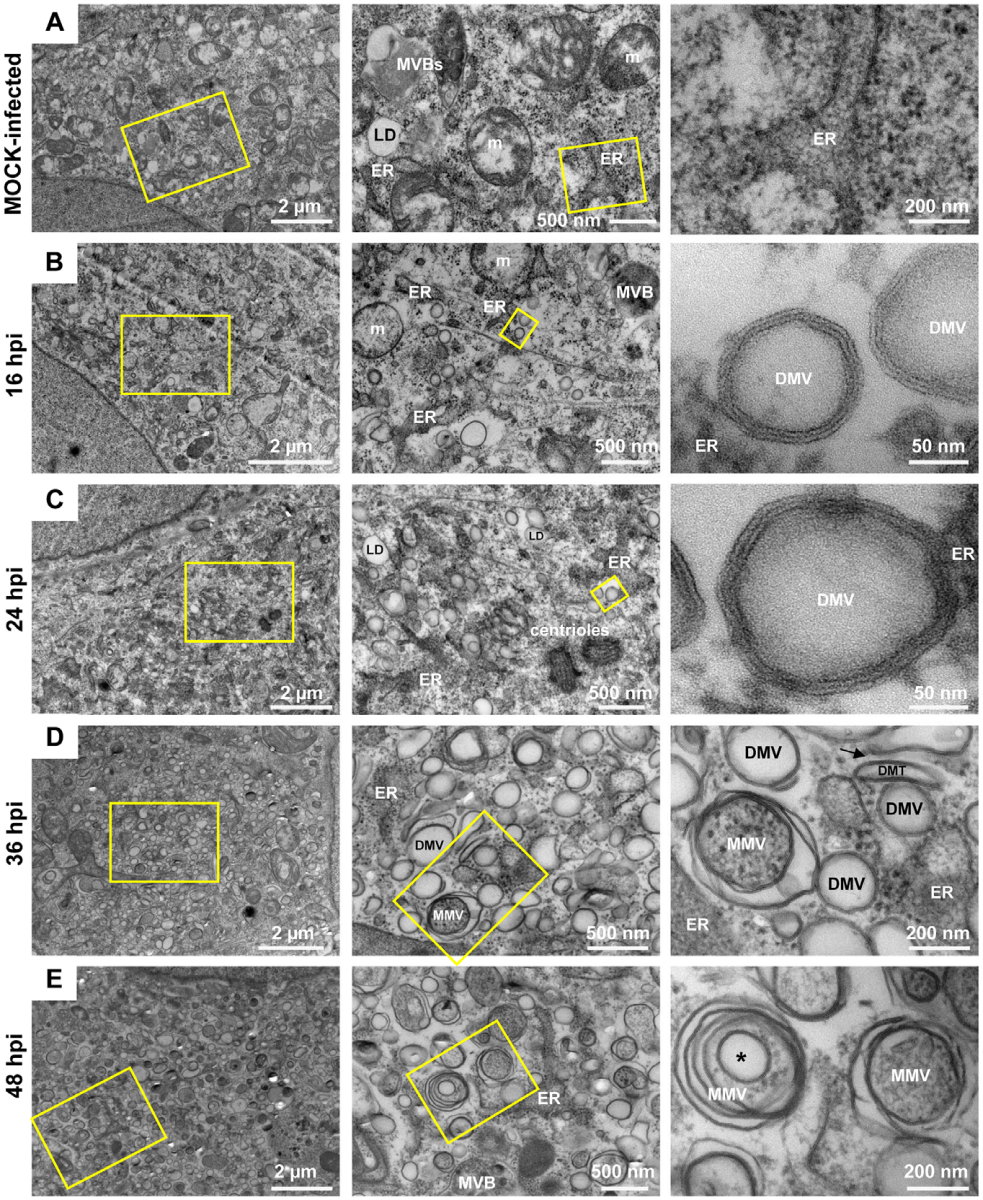
Time course of membrane alterations upon HCV infection. (A) Images of mock-infected high-pressure-frozen and freeze-substituted Huh7.5 cells. (B–E) Micrographs of high pressure frozen freeze-substituted Huh7.5 cells harvested 16, 24, 36 and 48 h after infection with Jc1 (100 TCID_50_/cell). Yellow squares indicate the areas that are shown at higher magnification on the right of each subpanel. Note the time-dependent increase of complexity of HCV-induced membrane alterations. ER, endoplasmic reticulum; m, mitochondria; MVB, multi-vesicular bodies; LD, lipid droplet; DMV, double membrane vesicle; MMV, multi membrane vesicle; DMT, double membrane tubule (labeled with a black arrow).

We then studied the kinetics of appearance of HCV-induced membrane alterations. Huh7.5 cells were infected with the highly assembly-competent HCV genome Jc1 [24], cells were fixed 4, 8, 12, 16, 24, 36 and 48 h post-infection (hpi) and processed for EM by using HPF-FS and epon embedding as described above. Mock-infected cells served as negative control (Figure 3A). At very early time points after infection (4–12 hpi), no virus-specific alterations of intracellular membranes were detected (not shown). At 16 hpi vesicles with two clearly distinguishable and closely apposed membrane bilayers were observed only in infected cells and these DMVs were the predominant structures (Figure 3B). They had an average diameter of ∼125 nm (±23 nm; n = 90) and resided primarily in the area surrounding rER cisternae suggesting that the DMVs originate from this compartment. At 24 hpi the number of DMVs per cell increased dramatically (for quantification see below) and their diameter was slightly larger (147 nm±26 nm; n = 90) (Figure 3C). At 36 and 48 hpi multi membrane vesicles (MMVs) became more predominant. They were composed of more than two bilayers, displayed in a concentric fashion and were abundant in the cytoplasm of late stage-infected cells (Figure 3D, E). The size of MMVs was much larger than the one of DMVs (337 nm±82 nm; n = 90). Some of the MMVs had an electron dense lumen, corresponding most likely to engulfed cytosol, while others appeared ‘empty’ arguing that they represent double membrane autophagosomes engulfing DMVs and cytosol (labeled with a * in Figure 3E). At these late time points after infection the cytoplasm was ‘filled’ with those vesicular structures giving the cytoplasm a sponge-like appearance. In addition, some of the DMVs displayed a larger tubular shape, which we therefore called double membrane tubules (DMTs).

We note that a locally confined accumulation of membranous vesicles originally referred to as MW was rarely observed when using HPF-FS of HCV-infected or replicon-containing cells, which is at variance to the study by Egger and co-workers who used conventional EM [18]. We only observed such confined sites when using expression of HCV proteins (see below). Nevertheless, in order to stay consistent with the generally accepted nomenclature, we use the term MW in the present report to indicate accumulations of HCV-associated membranous vesicles even though they were rarely confined to distinct subcellular areas under the used conditions.

### Localization of viral proteins to HCV-induced membrane structures

**Figure 4:**
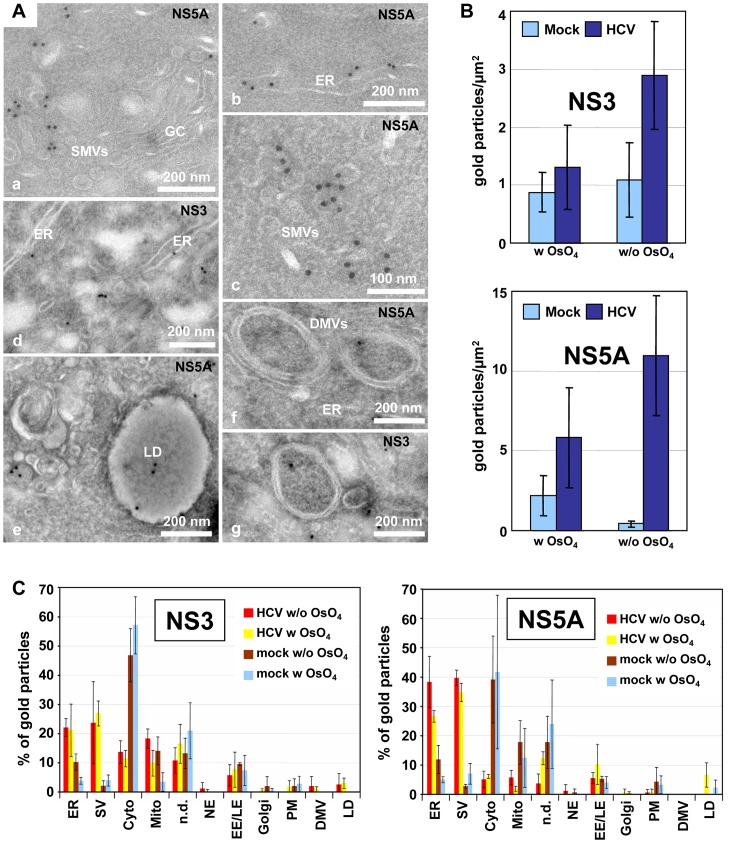
Immuno-EM localization of NS5A and NS3 in HCV-infected cells. Huh7.5 cells were infected with 100 TCID_50_/cell of Jc1 and fixed 16 h post-infection. (A) Cells shown in panels b, c and e to g were post-fixed with 0.1% OsO_4_ providing best membrane preservation, especially in case of DMVs (f and g) and LDs (e). Both NS5A and NS3 localize predominantly to the ER (b and d) and 50–70 nm diameter single-membrane vesicles (SMVs; subpanel a, c and d). NS5A also localizes to lipid droplets (e). (B) Amount of gold particles per µm^2^ in Jc1-infected versus mock-infected cells after immunolabeling with NS3- and NS5A-specific primary antibodies. Note the higher immunolabeling with samples prepared without OsO_4_, but also the lower membrane preservation under this condition. (C) Relative labeling distribution of NS3 and NS5A. Thawed cryosections of cells post-fixed or not with OsO_4_ were labelled with NS3- or NS5A-specific antibodies by using two different blocks and 3 different labeling experiments. Per labeling experiment two grids were considered, counting ∼100–200 gold particles per grid and attributing the particles to the indicated structures. In the case of uninfected cells only background labeling in the cytoplasm, on mitochondria and undefined structures was seen. Numbers refer to the percent of total gold particles counted per sample. ER, endoplasmic reticulum; SV, small vesicles; Cyto, cytosol; Mito, mitochondria; n.d., non-defined structures; NE, nuclear envelope; EE/LE, early/late endosomes; PM, plasma membrane; DMV, double membrane vesicle; LD, lipid droplet.

To determine localization of HCV proteins at distinct membranous structures, we performed immunolabeling of thawed cryo-sections. Surprisingly, conditions that we had used earlier to detect Dengue virus replication and assembly sites [9] and that supported high membrane preservation and antigenicity, failed in case of HCV-infected cells. After standard fixation and preparation of thawed cryo-sections for immunolabeling on cells 48 hpi, membranous areas appeared heavily extracted; membranes were no longer detectable and the cytoplasm appeared full of holes (not shown). Thus, to facilitate the analysis cells were fixed already 16 hpi, because at this time point HCV-induced membrane rearrangements were of rather low complexity and thus easier to preserve and interpret. Moreover, membrane preservation was increased when cells were post-fixed with osmium tetroxide (OsO_4_) [25], resulting in a much better overall structure preservation, although labeling efficiency was reduced by this method (Figure 4A, B). Of all antibodies tested we found that NS5A- and NS3-specific antibodies showed specific labeling on infected cells. The majority of the label was associated with small 50–70 nm diameter single membrane vesicles (SMVs) residing in close proximity of the Golgi complex (Figure 4Aa and 4Ac) and the rER (Figure 4Ab and 4Ad). Both antibodies also labelled DMVs (Figure 4Af and 4Ag), but to a much lesser extent. In addition, NS5A was found associated with LDs (Figure 4Ae) as described earlier [26].

Next we wanted to substantiate these data and determined the relative labeling distribution [27] by comparing infected to uninfected cells with and without OsO_4_ post-fixation. Results obtained in this way confirmed that irrespective of OsO_4_ post-fixation the structures most abundantly labelled by both NS5A- and NS3-specific antibodies were the rER and small vesicles, whereas in uninfected cells we detected only background labeling (Figure 4C). While the labeling on ER and Golgi-associated vesicles reflects the sites of synthesis (rER) and likely of accumulation, respectively, we were surprised to detect little HCV protein labeling on DMVs. For this reason we performed immunolabeling by using other EM-embedding methods (supplementary Figure S4A, Protocol S2 in Text S1), but conditions with high structure preservation were unfavourable for antigen detection and vice versa. Likewise, attempts to localize double-strand (ds) RNA by immuno-EM using a dsRNA-specific antibody were ambiguous (supplementary Figure S4B–F, Protocol S3 in Text S1). Even though in HCV-infected cells dsRNA labeling was higher as compared to mock-infected cells (supplementary Figure S4D), labeling could not unambiguously be allocated to a distinct membranous compartment (supplementary Figure S4E). The only exception were DMVs; ∼20% of these structures could be labeled either on the membrane or inside the vesicle and in ∼20% of cases the dsRNA label was in close proximity of DMVs (supplementary Figure S4B, F). However, attempts to corroborate these results by labeling HCV RNA metabolically were not successful, even though Dengue virus RNA could be detected with this method in infected Huh7 cells (not shown). In conclusion, the majority of HCV-specific antigen labeling resided at the ER and at small SMVs whereas DMVs were labeled with only low efficiency for HCV protein and dsRNA. These results are consistent with the important role of the ER for MW formation.

### Kinetics of DMV formation correlates with HCV RNA replication

**Figure 5:**
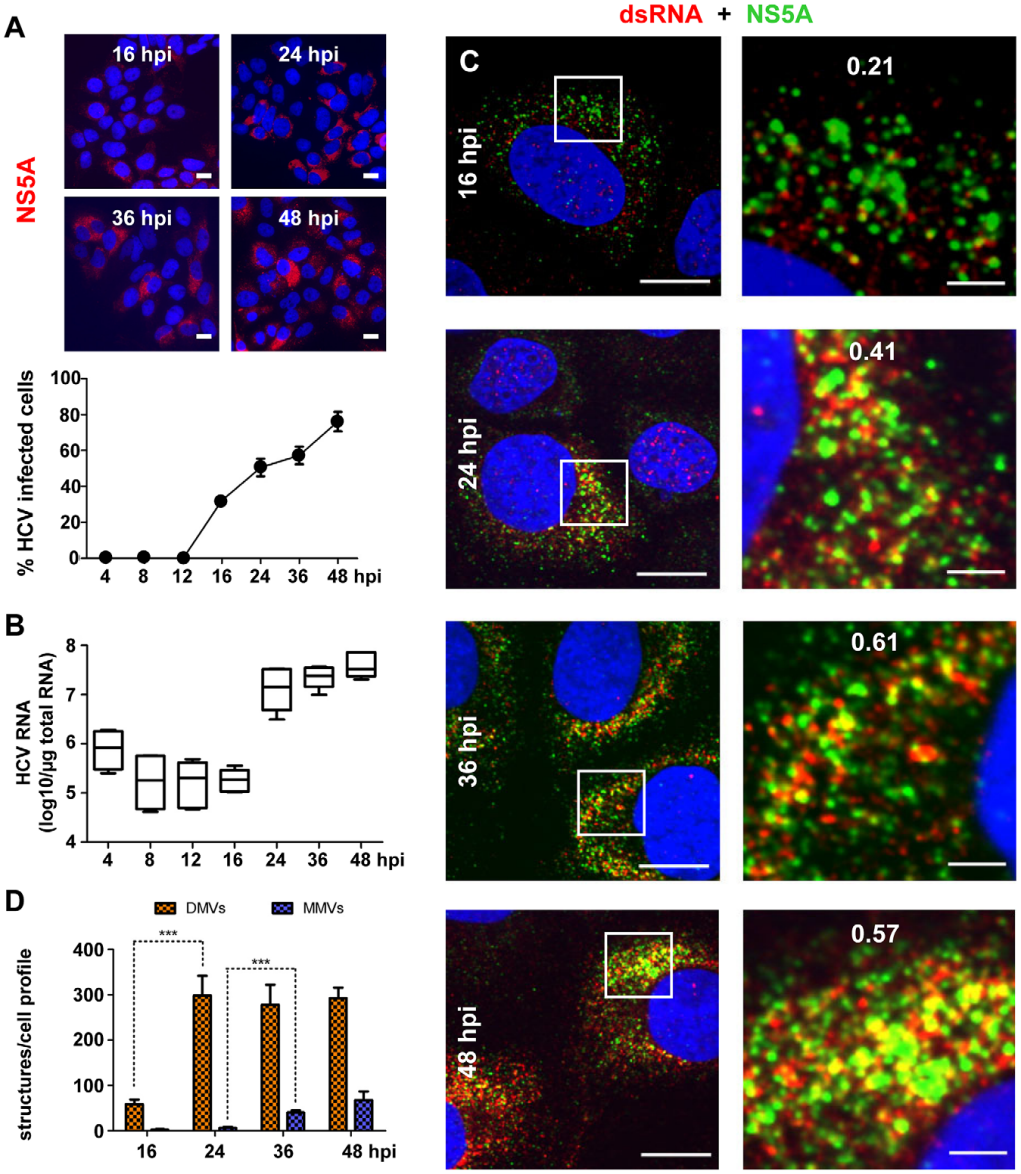
Correlation between HCV RNA replication and appearance of double membrane vesicles. (A) Time course of spread of HCV infection in Huh7.5 cells infected with 100 TCID_50_/cell of Jc1. Infected cells were detected by immunofluorescence using an NS5A-specific antiserum (upper panels). The graph shown below represents the result of counting ∼200 cells for each time point to determine the percentage of infected cells. Scale bars represent 50 µm. (B) Time course of accumulation of intracellular HCV RNA in infected Huh7.5 cells. The graph shows the result of two independent experiments (3 replicas each). Whiskers indicate the minimum and maximum values. (C) Colocalization of dsRNA and NS5A in cells infected with Jc1 (10 TCID_50_/cell). Cells were fixed at time points specified in the left of each panel row and NS5A and dsRNA were detected by indirect immunofluorescence microscopy. DNA was stained with DAPI (blue). Boxed areas in the left panels indicate areas that are shown as enlargements in the corresponding right panels. The quantification of the degree of colocalization (Pearson's correlation coefficient) is given in the enlarged pictures. Scale bars represent 10 µm and 2 µm (left and right panels, respectively). (D) Time course of accumulation of DMVs and MMVs. For each time point, 10 cellular profiles were counted. The Mann-Whitney (non-parametric) test was applied to determine statistical significance. Error bars refer to the standard deviation. Note the striking correlation between the increase of intracellular HCV RNA and DMV number between 16 and 24 hpi.

Given the difficulty to unambiguously detect viral RNA by immunolabeling methods, we used an alternative approach and determined whether the kinetics of RNA amplification correlated with the kinetics of membrane alterations. Huh7.5 cells were infected with HCV (clone Jc1) at 100 TCID_50_/cell and kinetics of spread of infection were determined by quantifying the number of NS5A-expressing cells during a time period of 4–48 h (Figure 5A). Infected cells were first detected 16 hpi and their number increased steadily thereafter. Owing to high amounts of input viral RNA, amplification of intracellular HCV RNA became first detectable 16 hpi and increased ∼100-fold during the subsequent 8 h (Figure 5B). This kinetic was corroborated when cells were analyzed by immunofluorescence microscopy to detect dsRNA, the presumed RNA replication intermediate, and its colocalization with NS5A, a replicase component (Figure 5C). We observed a time-dependent increase of the dsRNA- and NS5A-specific signals in Jc1-infected Huh7.5 cells, whereas no such signal was found in mock-infected control cells (not shown). Furthermore, the degree of colocalization between dsRNA and NS5A increased remarkably from ∼20% at 16 hpi up to ∼60% at 36 hpi, presumably as a result of dsRNA accumulation at replication sites. Importantly, quantification of the number of DMVs in these infected cells revealed an analogous rise in DMV abundance (Figure 5D). At 16 hpi only ∼50 DMVs were detected per cell section, but this number increased ∼6-fold during the following 8 h. Thereafter, DMV number per cell remained rather constant. In contrast, the number of MMVs was much lower throughout the observation period and their abundance increased only at later time points. Thus, the striking correlation between the kinetics of viral RNA amplification and DMV formation suggests that DMVs might play an important role for HCV RNA replication. In contrast, MMVs appeared in high abundance only after the exponential RNA amplification phase arguing that they are only of minor relevance for replication.

### Electron tomography identifies DMVs as protrusions from the ER

**Figure 6:**
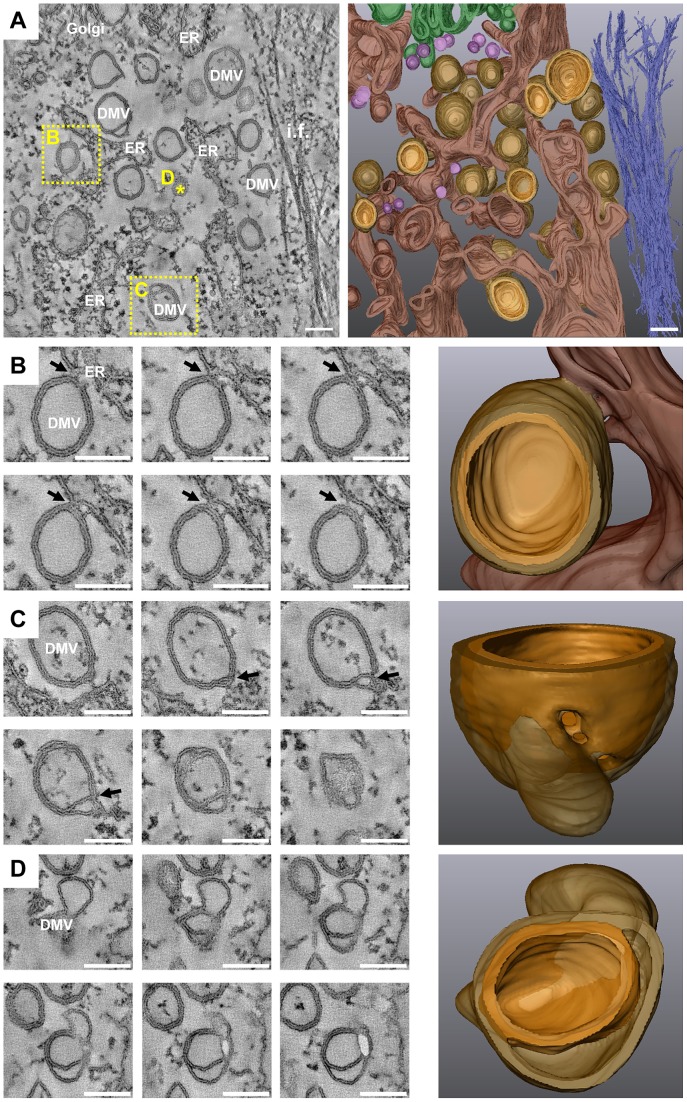
3D architecture of membrane rearrangements induced 16 h after HCV infection. (A) Huh7.5 cells were infected with 100 TCID_50_/cell of Jc1, fixed 16 h later and after HPF-FS processed for ET as described in materials and methods. Left: slice of a dual axis tomogram showing the various membrane alterations. Right: 3D reconstruction of the complete tomogram. Note the high number of DMVs. Panels B, C and D are part of the tomogram displayed in panel A and their position in panel A is highlighted by either a yellow dashed square (B and C) or by a star (D). In the 3D models shown on the right, the ER is depicted in dark brown, the inner membrane of DMVs and DMTs in yellowish brown and their outer membrane in semi-transparent light brown. Single membrane vesicles are colored in pink, intermediate filaments in dark blue and the Golgi apparatus in green. (B) Left: serial single slices through the same tomogram shown in panel (A) displaying a connection between the outer membrane of a DMV and the ER membrane (black arrows). Right: 3D surface model showing the membrane connection. (C) Left: serial single slices through the same tomogram illustrating a lasso-like structure of a DMV that after rendering reveals a pore-like opening that connects the interior of the DMV with the cytosol. The position of this opening in the 2D slice is marked with a black arrow. Right: 3D view of this DMV showing the ‘pore’. (D) Left: serial single slices through the same tomogram showing a DMV with a large inter-membrane space between its inner and outer membranes. Right: 3D view of this DMV. Scale bars represent 100 nm. This tomogram is shown in movie S1.

To gain insight into the 3D architecture of membrane alterations induced by HCV we carried out electron tomography (ET) analysis of 250 nm thick sections of Huh7.5 cells infected with Jc1 for 16 or 36 h. As shown in Figure 6, the outer (cytosolic) membrane bilayer of a large fraction of DMVs (∼45% of the vesicles that were fully included in the volumes of a total of 149 vesicles examined in 19 different tomograms at different time points) were continuous with the ER membrane. Surprisingly, in contrast to our earlier observations made with Dengue virus-infected Huh7 cells [9], HCV-induced DMVs appeared as exvaginations, connected via a short neck-like structure to the ER membrane bilayer. This is exemplified by the consecutive snapshots and the 3D surface rendering of a tomogram generated from cells analyzed 16 hpi (Figure 6B and supplementary Movie S1). The fact that these DMVs were linked to the rER only via their outer membrane suggests that these connections might represent an intermediate stage of DMV formation, eventually prior to their separation from the ER. In fact, ∼40% of the DMVs were tighly apposed to ER membranes, but without a visible neck-like structure whereas ∼15% of DMVs were not at all linked to ER membranes.

If DMVs would be sites of HCV RNA replication, viral replicase could either reside on the DMV surface, and thus oriented towards the cytosol as discussed e.g. for the poliovirus [28], [29] (see below), or in the interior of the DMV. In the latter case one would expect that the vesicle would have a pore to allow entry of e.g. NTPs and exit of viral RNA progeny. We therefore studied DMV morphology in more detail by ET and 3D reconstructions. Most of the DMVs appeared as closed structure and only in a minority of cases (∼8%) an opening (‘pore’) towards the cytosol was found (an example is shown in Figure 6C). Thus, either only a small fraction of DMVs is actively engaged in HCV RNA replication at a given time point or the viral replicase might reside on the surface of DMVs. We also noted that most DMVs have two tightly apposed bilayers. However, in a small fraction of DMVs an intermembrane space was observed (Figure 6D). Whether this represents another intermediate step in the formation of DMVs or is caused by sample preparation is not known.

**Figure 7:**
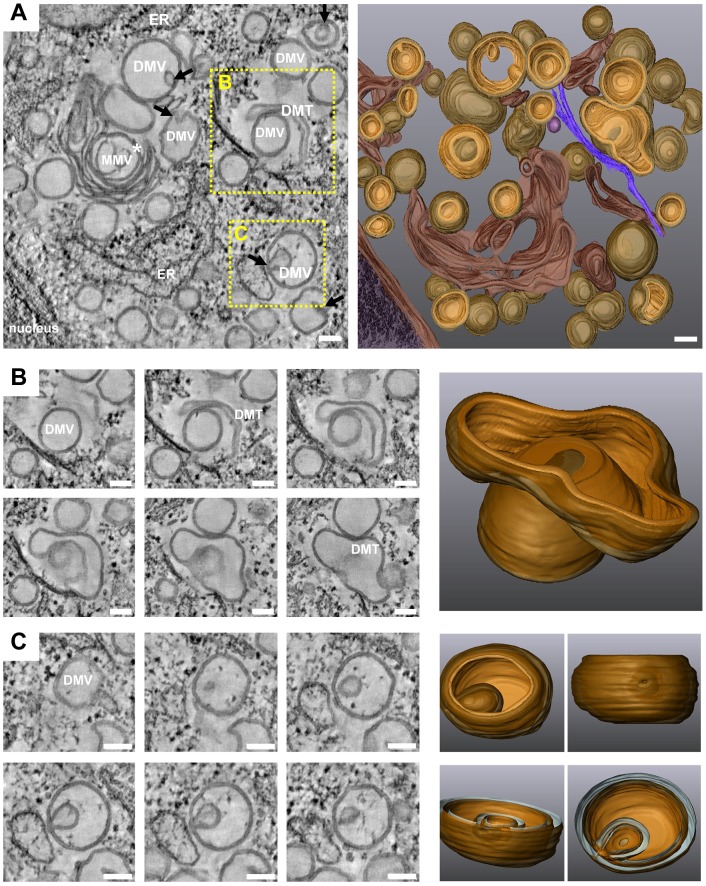
3D architecture of membrane rearrangements induced 36 h after HCV infection. (A) Left: slice of a dual axis tomogram taken from Huh7.5 cells 36 h after infection with 100 TCID_50_/cell of Jc1. Right: 3D reconstruction of the tomogram. Note the extensive membrane reorganization and the appearance of MMVs predominating at late time points after infection. Black arrows show invaginations of DMVs. The white star indicates a large MMV; due to its complexity only its middle part could be rendered. (B) Left: slices through the same tomogram highlighting a DMT enwrapping a DMV and presumably leading to the formation of a MMV. Right: 3D surface rendering of this structure. (C) Left: slices through the same tomogram highlighting a ‘self-invagination’ event of a DMV, also leading to the formation of a MMV. Right: 3D surface rendering of this late structure, revealing an opening of this MMV towards the cytosol as a result of the self-invagination. Panels B and C are part of the tomogram displayed in panel A and their positions are highlighted by yellow dashed squares in panel A. Scale bars represent 100 nm. For further details see legend to Figure 6. This tomogram is shown in movie S2.

Late during HCV infection, in addition to DMVs, MMVs and DMTs increased in abundance (Figure 7 and supplementary Movie S2), with the latter corresponding most likely to elongated versions of DMVs (Figure 7B). In fact, at 36 hpi numerous enwrapping events could be observed including DMVs containing smaller DMVs inside (Figure 7C). It is likely that the extensive enwrapping and curling of membranes leads to the formation of MMVs. Their formation might be due to high abundance of the viral proteins accumulating at the ER membrane at this late stage of infection or be caused by an autophagic host cell response. In any case, the late appearance of MMVs argues that they are not the major sites of HCV RNA replication.

### A concerted action of HCV replicase proteins is required for membranous web formation

**Figure 8:**
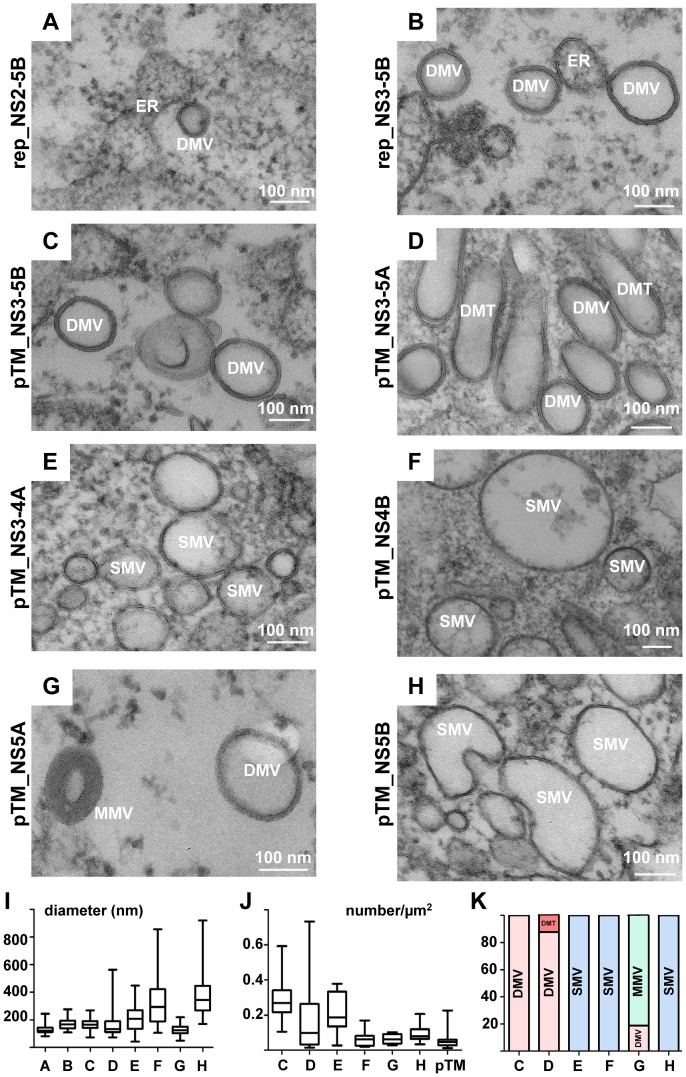
Membrane alterations induced by expression of individual HCV proteins. Huh7.5 cells transfected with replicon RNAs (A, B) or Huh7-Lunet-T7 cells transfected with pTM-based expression constructs (C–H) specified in the left of each panel, respectively, were high pressure frozen 24 h after transfection, freeze substituted, embedded into epon resin and sections were analyzed by transmission EM. Representative images showing HCV-induced membrane alterations are shown. (I) Average diameter of specific vesicular structures, either DMVs or SMVs, detected in cells that had been transfected with constructs specified in the upper panels. Whiskers represent minimum and maximum values. (J) Number of vesicular structures detected in profiles of 10 transfected cells. Cells transfected with the pTM expression vector without HCV insert were used as reference. The number of vesicular structures per µm^2^ is given; whiskers represent minimum and maximum values. (K) Relative abundance of membranous structures. Note that only upon expression of the NS3-5A polyprotein and NS5A two different structures were observed. In all other cases, only one membranous structure was detected.

With the aim to gain insight into the mechanism of HCV-induced membrane alterations, we first determined the contribution of individual viral proteins to this process. In the first set of experiments we analyzed intracellular membrane alterations detected in cells that had been transfected with subgenomic replicons encoding NS2 to 5B or NS3 to 5B polyprotein fragments, respectively (Figure 8A, B). In both cases DMVs were detectable resembling morphologically those found in HCV-infected cells. However, MMVs were virtually absent in replicon-transfected cells arguing that the structural proteins themselves or a stress response triggered by these proteins is the primary determinant for MMV formation. Moreover, cells transfected with the NS2-5B replicon contained lower numbers of DMVs, which correlated with its lower replication efficiency as compared to the NS3-5B replicon (not shown). The very same membrane alterations were found upon expression of a NS3-5B polyprotein fragment demonstrating that formation of DMVs is induced by viral proteins independent from HCV RNA replication (Figure 8C; quantified in panels I–K). Interestingly, expression of a NS3 to 5A polyprotein fragment lacking NS5B induced the formation of small clusters of DMVs and elongated double membrane tubules (DMTs) (Figure 8D) having a highly variable diameter (166 nm±92 nm; n = 90; Figure 8I). This result indicates that apart from its role as RNA-dependent RNA polymerase, NS5B also affects morphology of the MW. Nevertheless, formation of DMVs does not require this HCV protein.

To study the contribution of individual HCV proteins to the formation of the MW, we expressed NS3/4A, NS4B, NS5A or NS5B proteins separately in Huh7-Lunet cells and assessed by EM membrane alterations in cell sections after epon embedding. We did not express NS3 and NS4A on their own, because both proteins form a stable membrane-associated complex and only this complex is physiologically relevant [30]. In agreement with an earlier report [18], in cells expressing the NS3/4A complex, we detected swollen ER sheets as well as SMVs of variable diameter (203 nm±93 nm; n = 90) (Figure 7E and I–K). Strikingly, the individual expression of NS4B (Figure 8F) induced the formation of only SMVs (325 nm±168 nm; n = 90) whereas DMVs were not observed (Figure 8K) even though NS4B is considered to be the main inducer of the MW. To our great surprise, in cells expressing NS5A we observed curling of ER membranes and formation of MMVs containing several lipid bilayers in a concentric manner and with an average diameter of 125 nm (±35 nm; n = 90) (Figure 8G, K). Interestingly, some of these vesicles displayed only 2 lipid bilayers and their morphology was indistinguishable from DMVs observed in HCV-infected cells or cells containing a subgenomic replicon. Expression of NS5B induced enlarged ER sacs with an average diameter of 370 nm (±150 nm; n = 90) and occasional curvature (Figure 8H and I–K). In summary, none of the HCV proteins was capable on its own to induce formation of the MW, which requires the concerted action of two or more replicase proteins. Importantly, NS5A was the only protein inducing the formation of DMVs arguing that NS5A is a major contributor to MW biogenesis.

## Discussion

In this study we conducted a detailed analysis of the 3D morphology and biogenesis of the intracellular membrane structures induced by HCV. Our results have several implications for our understanding of the HCV replication cycle and its relation to replication strategies of other positive-strand RNA viruses.

### Origin of HCV-induced membrane rearrangements

By using an extensive IF-based analysis, we demonstrate that the main component of the HCV-induced MW is membranes derived from the ER. Moreover, we detected markers of early and late endosomes, COP vesicles, mitochondria and LDs. These results support earlier studies showing the association of the MW with ER membranes and LDs [19], [31], [32]. Although we observed colocalization of HCV proteins with mitochondria, at variance to previous reports we did not detect morphological alterations of mitochondrial membranes as compared to uninfected cells [33]. We also detected colocalization of HCV proteins with Rab proteins found in early and late endosomes and these proteins have been identified as host factors required for HCV genome replication [34]–[36]. Since they are involved in regulating vesicle budding, transport and fusion with target membranes we assume that endosomes are involved in the trafficking of NS proteins or viral RNA to distinct sites within the MW such as sites of RNA translation, replication or virus assembly/exit. In fact, by using immunolabeling we found that NS5A and NS3 localize to SMVs, that were found in close proximity to the Golgi complex and the ER, hence representing potential COP vesicles. Likewise, we observed COP I vesicles in close proximity of DMVs and LDs (not shown) supporting the important role of COP I components and lipid/LD homeostasis for HCV replication [37].

A recent report by Ferraris and co-workers described the detection of LC3-II at HCV-induced membranes [20] arguing that formation of HCV replication sites is linked to autophagy. However, the role of autophagy in the HCV replication cycle is a matter of controversy. It has been proposed that autophagy is involved in HCV RNA translation [38], initiation of RNA replication [39], [40], production of infectious virus particles [41] or suppression of the innate antiviral defense [42], [43]. While autophagy has been shown to play a major role for the replication of several other positive-strand RNA viruses such as poliovirus [44], [45], coronaviruses [46] or Dengue virus [47], we did not detect colocalization of HCV proteins with LC3 (expressed endogenously or as GFP-LC3 fusion protein). While this observation is in complete agreement with reports by others [38], [39], [41], [48] it is well possible that individual factors of the autophagy pathway, rather than the complete machinery, are involved in the formation of the MW. Indeed it has been recently reported that NS4B forms a complex with Rab 5 and Vps34 and induces autophagy [49]. Moreover, NS5B appears to interact via its thumb domain with Atg5 [40]. Interestingly, Atg5 initiates the formation of DMVs via a crescent shape membrane and colocalizes with NS5B [40]. It is therefore tempting to speculate that proteins of the autophagy machinery, in close collaboration with HCV proteins, induce formation of DMVs. Based on our observation that NS5A triggers DMVs and MMVs and the recent finding that NS5A is sufficient for induction of autophagy and contributes to the fusion of autophagosomes and lysosomes [50], we assume that NS5A plays a key role in the biogenesis of membrane rearrangements.

In addition to DMVs, we also observed MMVs in HCV-infected cells. The late appearance of these membrane structures upon HCV infection argues against a direct role in RNA replication. Instead MMVs might be an epiphenomenon induced e.g. by a stress response potentially caused by massive membrane rearrangements [49], [51] or by the high abundance of membrane-associated HCV proteins [52], [53].

### Biogenesis of DMVs and their possible role for HCV RNA replication

The predominant membranous structures detected in HCV-infected cells were DMVs. These structures as well as MMVs were not an artifact caused by the used methods because they were consistently detected under the following conditions (supplementary Figure S5):

- with naïve Huh7 cells infected with Jc1, thus excluding cell clone-specific effects (Figure S5A);
- with cells transiently transfected with a subgenomic JFH-1 (genotype 2) replicon and processed for EM analysis without chemical fixation, thus excluding fixation artifacts (Figure S5B);
- with cells containing a stably replicating genotype 1b replicon thereby excluding genotype-specific effects (Figure S5C);
- with cells infected with low MOI and harvested at different time points after infection (Figure S5D, E).

Thus, DMVs and MMVs are membrane rearrangements that occur independent from the mode of viral RNA delivery and fixation method and that do not depend on HCV genotype, a particular cell clone, chosen MOI and time point of cell harvest.

By using single protein expression we found that each HCV replicase factor induced distinct membrane alterations. In case of the NS3/4A complex large SMVs were found whereas expression of NS4B induced smaller and more homogenous SMVs. Importantly, expression of NS5A led to the formation of DMVs and MMVs whereas NS5B induced enlargements of the ER occasionally containing invaginations. Interestingly, by using cell lines inducibly expressing single HCV replicase proteins in U-2 OS human osteosarcoma cells, Egger and co-workers observed analogous membrane alterations in NS3/4A- and NS4B-expressing cells that corresponded to smooth SMVs and compact clusters of vesicles, the latter called MW [18]. However, no membrane alterations were detected in cells over-expressing NS5A or NS5B. Importantly, in cells expressing the complete HCV polyprotein, in addition to the smooth SMVs and the membranous web, so-called contiguous vesicles were found, which could not be ascribed to individual HCV protein(s) [18]. These contiguous vesicles were of irregular shape and their membranes were in very close contact in a way that the two lipid bilayers were often fused into a trilayer. These vesicles thus resemble DMVs that we observed in HCV-infected cells or in cells expressing NS5A. The reason why such vesicles have not been observed by Egger and co-workers in cells expressing only NS5A is not known, but might be due to the use of different expression systems or different conditions of sample preparation.

**Figure 9:**
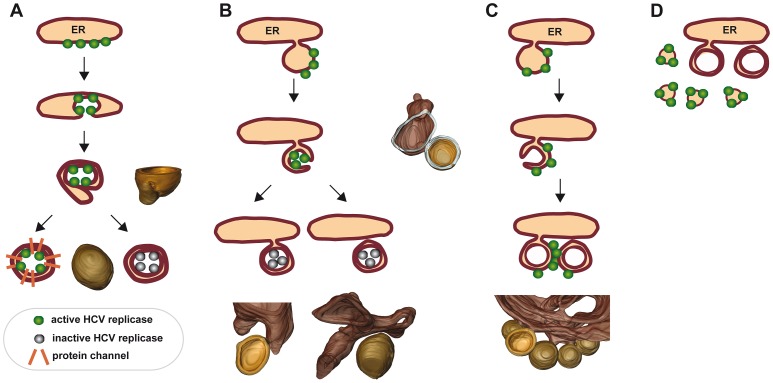
Hypothetical models describing the formation of double membrane vesicles and their possible role in viral RNA replication. (A) By analogy to flaviviruses [9] HCV proteins induce invaginations of the ER membrane. Extensive invagination leads to a local ‘shrinking’ of the ER lumen. This model assumes that enzymatically active HCV replicase (green dots) reside in the lumen of the invagination and remain active as long as the vesicle is linked to the cytosol. Upon closure of the DMV, the replicase would become inactive (grey dots). Alternatively, closed DMVs might be connected to the cytosol via proteinaceous channels. (B) HCV proteins might induce tubulation of ER membranes that undergo secondary invagination and thus double membrane wrapping. These DMVs could initially be open to the cytosol, but might close off as replication/infection progresses. The resulting DMV might stay connected to the ER via a stalk or be released as a ‘free’ DMV (left or right drawing, respectively). (C) Induction of DMVs follows the same pathway as described for panel B, but the viral replicase remains on their cytosolic surface as discussed e.g. for the poliovirus [28], [29]. (D) HCV RNA replication might occur on SMVs in close proximity of DMVs. In this case, DMVs might be an epiphenomenon or serve some other purpose for the HCV replication cycle. For each model, structures identified in the 3D reconstructions are shown next to or below the corresponding schematic drawing. For further details see text.

So far we can only speculate about the biogenesis of DMVs. In one scenario, HCV proteins might provoke membrane invaginations into the ER, similar to what we had described for the related Dengue virus [9]. Such invaginations might enlarge and reach the opposite ER membrane, thus leading to pairing of both lipid bilayers and DMV formation (Figure 9A). In a variation of this model, HCV proteins would induce local exvaginations from the ER membrane leading to vesicles that might remain linked to the ER via a short membranous stalk (Figure 9B). These initially single-membrane vesicles might undergo a secondary invagination to form DMVs. Consistent with this second model we detected a significant proportion of HCV proteins at small SMVs (Figure 4). Moreover, in this model DMVs with a ‘pore’ (Figure 6C) might represent intermediates that are (still) actively involved in RNA replication, whereas later on when DMVs become completely sealed, they would represent remnants that are no longer actively engaged in the RNA amplification process. Such a compact structure might also shield HCV proteins and RNA from detection by antibodies, which would explain the poor immunolabeling of DMVs (Figure 4). Alternatively, one might hypothesize that the HCV replicase resides on the cytosolic surface of DMVs analogous to what is assumed for the poliovirus [54] (Figure 9C). Although counter intuitive, also in this case viral RNA and proteins actively engaged in RNA replication are shielded from degradative enzymes, at least to some extent, presumably by tight clustering of replication vesicles and positioning of the replicase towards the center of vesicle clusters [54].

Clearly, a direct demonstration of the HCV replication site will require the unequivocal detection of newly synthesized RNA. Detection by dsRNA-specific antibody is not sufficient, because this method does not discriminate between active and inactive, dsRNA-containing replication complexes. It might also detect viral RNA genomes containing secondary (ds) structures and engaged e.g. in RNA translation or virion assembly. Unfortunately, several attempts to detect newly synthesized HCV RNA by IF- or EM-based methods were not successful (not shown). These included (i) transfection of infected cells with 5-bromouridine 5′-triphosphate and subsequent detection with a bromodeoxyuridine-specific antibody; (ii) the use of chemically modified nucleotides (5-ethynyl uridine) suitable for detection by click-chemistry; (iii) the use of correlative microscopy after dsRNA-specific immunofluorescence. These failures are probably due to poor accessibility of viral RNA to antibodies, the fragile nature of the MW and the rather low replication of HCV. In support of this assumption, some of these approaches allowed detection of newly synthesized Dengue virus RNA that replicates to much higher levels in an easy to access ER-derived membrane compartment (Lee et al., unpublished) [9].

We note that for HCV most immunolabeling was detected at the ER and SMVs whereas DMVs were only sporadically labeled (Figure 4 and supplementary Figure S4B). While labeling of the ER is consistent with its role as primary source of the MW, the role of SMVs in the HCV replication cycle is unknown. On one hand they might serve as sites of viral RNA replication (Figure 9D) whereas DMVs might be an epiphenomenon reflecting e.g. a stress response. On the other hand, DMVs might be sites of HCV replication (Figure 9 A–C) and their poor immunolabeling could be due to poor accessibility of viral RNA and proteins to antibodies; in this scenario SMVs might represent an epiphenomenon. In any case, in the absence of a robust method allowing metabolic labeling of viral RNA, the exact site of HCV RNA replication within the MW remains to be determined.

### Comparison of HCV-induced membrane alterations with those of other positive-strand RNA viruses

A common denominator of all positive-strand RNA viruses is the induction of membranous replication compartments that provide a physical scaffold for the assembly of macromolecular complexes catalyzing the amplification of the viral RNA genome [8]. Both the origin and the biogenesis of these compartments differ very much between the virus groups. For instance, Flock House virus induces ∼50 nm diameter invaginations of the outer mitochondrial membrane [11]. Plant pathogens like Tomato Bushy Stunt virus induce a remodeling of peroxisomes and chloroplasts [55]. The Semliki Forest Virus triggers exvaginations (spherules) at the plasma membrane [12]. Spherule-containing vesicles are internalized and fuse with acidic endosomes. As a final result, spherules accumulate on the outer surface of large vacuoles in the pericentriolar region. Even more complicated structures are induced by severe acute respiratory syndrome (SARS) coronavirus and Equine Arterivirus. Structures induced by these viruses are composed of mixtures of convoluted membranes and large (200–400 nm) DMVs [15]–[17]. In case of the Dengue virus belonging to the same virus family as HCV, invaginations of ER membranes are induced that are connected to the cytosol via 11 nm diameter pores allowing exit of viral RNA [9]. Similar observations have been made for another flavivirus, the West Nile virus [10]. Finally, poliovirus extensively reorganizes membranes originating from components of the anterograde membrane traffic system, giving rise to 50–400 nm single and double-membrane vesicles forming a complex meshwork [56], [57]. A recent study by Belov and co-workers proposed that poliovirus-induced DMVs are derived from SMVs that undergo complex secondary invaginations and enwrapping events [13]. In these respects, the HCV-induced MW morphologically most closely resembles the membranous replication compartment induced by poliovirus and members of the order *Nidovirales* (coronaviruses and arteriviruses). This similarity might reflect the use of common host cell pathways such as the phosphatidyl-inositol (PIP) pathway that plays an essential role in the formation and integrity of the membranous replication sites of HCV and picornaviruses [58]–[60]. Interestingly, morphological similarities also exist between the MW of HCV and the replication compartment of arteriviruses [17]. It would therefore be interesting to determine whether also this virus group utilizes PI4-kinases to establish its replication site.

In conclusion, we describe the first 3D model of HCV-induced membrane alterations that are associated with viral RNA replication. The biogenesis and morphology of the MW reveals an unexpected similarity to the distantly related picornaviruses, coronaviruses and arteriviruses. We propose that this similarity reflects the common use of host cell pathways for biogenesis and functionality of the membranous structures induced by these viruses.

## Materials and Methods

### Antibodies

Primary antibodies used for detection of HCV proteins or cellular proteins are specified in Table S1 in Text S1. Immunofluorescence analysis was performed using goat secondary antibodies conjugated with AlexaFluor 568 and Alexa Fluor 488 (Molecular Probes, OR, USA). Cellular DNA was stained with 4′, 6′-diamidino-2-phenylindole dihydrochloride (DAPI; Molecular Probes). Lipid droplets were visualized by staining with BODIPY 493/503 (Molecular Probes) and mitochondria were staining using MitoTracker Red (Molecular Probes).

### Cell culture

For virus production, infection assays and electroporation of HCV-RNA we used the cell clone Huh7.5 that is derived from the human hepatoma cell line Huh7 and that is highly permissive for HCV RNA replication [61]. Owing to unfavorable morphology of Huh7.5 cells, for all immunofluorescence assays we used high-passage naïve Huh7 cells that also efficiently support HCV replication and virus production. Huh7-Lunet cells [62], another highly permissive Huh7 subclone, was employed for electroporation of subgenomic HCV replicon RNAs [23], [63]. The use of these replicons allowed preparation of cells without prior chemical fixation and thus virus inactivation. Huh7-Lunet T7 cells were cultured in the presence of 5 µg of zeocin/ml and used for transfection with pTM-based expression plasmids [64]. Cells were grown in Dulbecco's modified Eagle medium (DMEM; Life Technologies, Karlsruhe, Germany) supplemented with 2 mM L-glutamine, nonessential amino acids, 100 units penicillin per ml, 100 µg streptomycin per ml and 10% fetal calf serum (DMEM complete).

### *In vitro* transcription and RNA transfection

PFK-based plasmids pFK-J6/C3 (Jc1), pFK-I389-neo-sg-JFH1, pFK-I389-Luc-NS2-3′_JFH_δg, pFK-I389Luc-NS3-3′_JFH_δg, the non-replicative mutant pFK-I389-Luc-NS3-3′-NS5BΔGDD_JFH_δg, pFKi389LucNS3-3′_dg_JFH-1_NS5Aaa2359_emGFP and the full-length genome pFK-JFH-δg have been described elsewhere [23], [24], [63], [65]. Synthesis of *in vitro* transcripts and RNA transfection by electroporation has been described in detail elsewhere [66].

### Preparation of virus stocks

Huh7.5 cells were transfected by electroporation with Jc1-derived *in vitro* transcripts. Culture supernatants of transfected cells were harvested 24, 48, 72 and 96 h after transfection and cleared by passing through 0.45-µm pore size filters. Supernatants were pooled and concentrated by using Centricon Plus-70 Centrifugal Filter Units (Millipore). Concentrates were loaded on top of an 80% Optiprep (Axis-Shield, Oslo, Norway) cushion in PBS and centrifuged for 4 h at 4°C and 30,000 rpm in an SW 28 Ti rotor (Beckman Coulter, Krefeld, Germany). Alternatively, supernatants were precipitated by using PEG-8,000 as described elsewhere [67]. Precipitate was spun down at 8,000× g for 90 min and resuspended in complete DMEM. Concentrated virus sample was collected and infectivity titer was determined by limiting dilution assay.

### Transfection of DNA expression constructs

GFP-Rab7, GFP-Rab11, GFP-Rab21 or YFP-β-COP expression constructs (kindly provided by Jeremy Simpson, EMBL, Heidelberg) or the GFP-LC3 construct (kind gift from Nathan Brady, Bioquant, Heidelberg) were transfected into Huh-7 high passage cells. A total of 0.2 µg DNA was transfected into cells seeded onto coverslips by using the Effectene transfection reagent (Qiagen, Hilden, Germany) according to the instructions of the manufacturer. Twenty-four hours later cells were analyzed by epifluorescence microscopy and inoculated with 30 TCID_50_/cell of Jc1 as described above. pTM vectors allowing expression of JFH1-derived polyprotein fragments or individual proteins [68] were transfected by using the Mirus TransIT-LT1 Transfection Reagent (Mirus Bio LLC) according to the instructions of the manufacturer. Twenty-four hours later cells were analyzed by EM.

### Quantification of viral RNA

Total RNA from ∼6×10^5^ HCV-infected or mock-treated Huh7.5 cells was isolated by using the Total RNA Isolation Nucleospin RNA II Kit (Macherey-Nagel, Düren, Germany) according to the recommendations of the manufacturer. HCV RNA was quantified by qRT-PCR using the One-step RT-PCR Kit (Qiagen, Hilden, Germany). Briefly, 5 µl of isolated RNA was added to a reaction mixture, containing 0.6 µl of enzyme mixture, 25 mM MgCl_2_, 100 µM of each primer (sense primer: 5′-TCTGCGGAACCGGTGAGTA-3′; antisense primer: 5′-GGGCATAGAGTGGGTTTATCC-3′), 10 mM of each dNTP and 10 µM HCV-specific probe (5′-6FAM (6-Carboxy-Fluorescine)- AAAGGACCCAGTCTTCCCGGCAATT- TAMRA (Tetra-Chloro-6-Carboxy-Fluorescine)-3′). Serial dilutions of a standard RNA (10^8^ to 10^2^ HCV RNA copies per reaction) were processed in parallel to determine absolute RNA amounts. Reactions were performed on a PRISM 7000 Sequence Detection System (Applied Biosystems, Darmstadt, Germany) using the following program: 50°C for 30 min, 95°C for 15 min and 40 cycles as follows: 94°C for 15 s, 55°C for 30 s and 72°C for 30 s. HCV RNA amounts in infected cells were normalized to the signal obtained with RNA from mock-treated cells.

### Immunofluorescence microscopy

Cells were seeded onto glass coverslips in 24-well plates and infected with 30 TCID_50_/cell of Jc1. At time points specified in the text cells were fixed with 3% paraformaldehyde (PFA; EM grade, Electron Microscopy Sciences, PA, USA) for 15 min. In case of detection of dsRNA, cells were fixed with methanol for 10 min at −20°C. After washing with PBS cells were permeabilized with 0.01% Digitonin or 0.5% TritonX-100 in PBS, washed with PBS and incubated for 10 min in PBS containing 1% BSA. Cells were incubated with primary antibody dissolved in PBS/1% BSA for 1 h at room temperature, washed extensively with PBS and incubated with Alexa Fluor-conjugated secondary antibody (diluted 1∶1,000) for 1 h at room temperature in the dark. After washing with PBS, DAPI stain (diluted 1∶5,000) was added and cells were incubated 1 min in the dark. Cells were washed with PBS and water, mounted with Vectashield (Vector Laboratories Inc., Burlingame, USA) and sealed with nail polish. For image analysis we used a Perkin Elmer Ultraview ERS spinning disk on a Nikon TE2000-E inverted confocal microscope equipped with a Plan-Apochromat VC 60× objective (NA 1.20) and the Volocity 5.3 software package. Channels were recorded sequentially onto an EM-CCD camera using 405 nm excitation and 445/460 nm emission for DAPI, 488 nm excitation and 527/555 nm emission for BODIPY and Alexa Fluor 488, and 568 nm excitation and 615/670 nm emission for Alexa Fluor 568 and MitoTracker. Images were merged by using the ImageJ software package (National Institutes of Health).

### High pressure freezing and freeze substitution (HPF-FS)

Cells were seeded onto 3 mm sapphire discs (M. Wohlwend GmbH, Sennwald, Switzerland) that had been carbon coated to improve cell adhesion. At different time points after infection (Jc1, 100 TCID_50_/cell) or electroporation of HCV RNA (10 µg) cells were fixed with 4% PFA, 0.1% GA in Na-cacodylate buffer [pH 7.4] and subsequently frozen after immersion in 1-hexadecene (Merck, Hohenbrunn, Germany) using a high-pressure freezer (M. Wohlwend GmbH). Frozen discs were stored in liquid nitrogen until further processing. Freeze substitution was done in acetone containing 0.2% (w/v) OsO_4_, 0.1% (w/v) UA, and 5% (v/v) water by slowly warming the samples from −90°C to 0°C during a period of 20 h [69]. Samples were kept at 0°C and at room temperature for 30 min each, washed with acetone, and embedded in two-step epon series (Fluka, Buchs, Switzerland) using 1 h-incubation in 50% epon dissolved in acetone and overnight incubation in 100% epon. Epon was exchanged, polymerized for 3 d at 60°C and sapphire discs were removed by immersion in liquid nitrogen. In case of cell pellet embedding that we used for immunolabeling (see Protocol S3 in Text S1), infected cells were scraped off the plate, pelleted by centrifugation, resuspended in 20% dextran, subjected to HPF-FS as described above and embedded into the methacrylate resin Lowicryl HM20, [Polysciences Inc., PA, USA]) that is more suitable for immuno-gold labeling. Resins were polymerized by treatment with UV light for 4 days.

### Immunolabeling of thawed cryo-sections

Infected cells were fixed by adding an equal amount of 8% PFA and 0.2% GA in 0.2 M PHEM buffer (120 mM Pipes, 100 mM Hepes, 4 mM MgCl_2_, 40 mM EGTA, pH 6.9) to the culture medium for 1 h at room temperature. Cells were then fixed for 1 h with 4% PFA, 0.1% GA and 1% Acrolein in 0.1 M PHEM at room temperature. The fixative was removed and the cells were stored at 4°C in 4% PFA in 0.1 M PHEM until further processing. After extensive washing with 0.1 M PHEM and alternative fixing with 0.1% osmium tetroxide for 30 minutes on ice [25], remaining aldehyde groups were blocked with 30 mM glycine in 0.1 M PHEM. Cells were scraped off the plate, embedded in 10% gelatine and infiltrated in 2.3 M sucrose overnight at 4°C. Cell pellets were mounted onto sample holder pins, frozen and stored in liquid nitrogen. 60 nm cryo-sections were prepared using a Leica Ultracut UC6 microtome (Leica Microsystems, Wetzlar, Germany) and a diamond knife (Diatome, Biel, Switzerland). Sections were picked up with a mixture of 2% methylcellulose and 2.3 M sucrose (1∶1) and after thawing transferred to 100 mesh formvar and carbon-coated grids. Labeling of thawed cryosections was performed essentially as described elsewhere [70]. In brief, sections were molten by floating on 2% gelatine for 30 min at 37°C, incubated in 30 mM glycine in 0.1 PHEM for 10 min and then in blocking solution (PBG: 0.8% [w/v] BSA [Sigma], 0.1% [w/v] fish skin gelatin [Sigma] in PBS). Sections were then incubated with primary antibody diluted in blocking buffer, for 30 min at room temperature. After 5 times washing (5 min each) in blocking buffer, sections were incubated with rabbit anti-mouse followed by protein A coupled to 10 nm gold (Cell Microscopy Center, Utrecht, The Netherlands) diluted in blocking solution. After washing with PBS and distilled water, grids were stained with 3% UA for 10 min at room temperature and incubated with a mix of 2% methylcellulose and 3% UA (1∶6) for 10 min on ice. The relative labeling distribution was determined essentially as described somewhere else [27].

### Correlative light-electron microscopy

Huh7-Lunet cells were transfected with 10 µg *in vitro* transcripts derived from the subgenomic replicon construct pFKi389LucNS3-3′_dg_JFH-1_NS5Aaa2359_emGFP [27] and seeded onto sapphire discs (M. Wohlwend GmbH) carbon coated with a finder grid (Electron Microscopy Sciences, Hatfield, Philadelphia, USA) on top to create a pattern. Twenty-four hours later, live cells were analyzed by fluorescence microscopy using a Zeiss Observer.Z1 inverted microscope (Carl Zeiss Microscopy GmbH, Germany) to identify cells containing the replicon and to record their positions in the patterned discs. Cells were then immediately fixed by HPF-FS and embedded into epon resin as described above. A small area around the recorded region of interest was trimmed and 60 nm thick serial sections were collected on formvar-coated slot grids for EM analysis. EM micrographs of GFP-positive areas were acquired at different magnifications to correlate these areas with the corresponding ultrastructural features. Images were taken on a Phillips CM 120 Biotwin microscope.

### Electron tomography

Sections of 250 nm thickness were collected on palladium-copper slot grids (Science Services, Munich, Germany) coated with Formvar (Plano, Wetzlar, Germany). Protein A-gold (10 nm) was added to both sides of the sections as fiducial markers. Dual axis tilt series were acquired with a FEI TECNAI F30 microscope operated at 300 kV and equipped with a 4 k FEI Eagle camera [binning factor 2, binned pixel size 0.592 nm (39.000× for the 16 hpi sample) or 0.998 nm (23.000× for the 36 hpi sample) on the specimen level] over a −65° to 65° tilt range (increment 1°) and at an average defocus of −0.2 µm. Tomograms were reconstructed using the weighted back-projection method implemented in the IMOD software package (version 3.11.5) [71]. Rendering of the 3D surface of the tomograms was performed by using the AMIRA Visualization software Package (version 5.2.2, Visage Imaging, Berlin, Germany). Models were generated from unfiltered and 2× binned tomograms by manually masking areas of interest, thresholding and smoothing labels.

## Supporting Information

Figure S1 **Colocalization of HCV proteins with cellular marker proteins.** Huh7 cells were infected with HCV (clone Jc1) using 30 TCID_50_/cell and 48 h later cells were fixed and processed for fluorescence microscopy. Detected HCV proteins are specified in the top of each subpanel, cellular proteins are given in the left of each panel. Upper panels represent a low magnification overview; boxed areas are shown as enlargement in the corresponding panel below. (A)–(C) Colocalization of HCV proteins with mitochondria stained with MitoTracker, lipid droplets labeled with ADRP, or COP II vesicles labeled with sec13, respectively. (D) Cells were transfected with a GFP-LC3 expression construct and 24 h later cells were infected as described above. DNA was stained with DAPI (blue). Samples were analyzed with a Nikon TE2000-E inverted confocal microscope at 60× magnification. Scale bars represent 10 µm (top panels) and 2 µm (lower panels). Representative images are shown. The quantification of the degree of colocalization (Pearson's correlation coefficient) is given in the enlarged pictures. N.a., not applicable due to cross-reactivity of antibodies.
(TIFF)

Figure S2 **Colocalization of HCV proteins with cellular marker proteins 24 h after infection.** Huh7 cells were infected with HCV (clone Jc1) using 30 TCID_50_/cell and 24 h later cells were fixed and processed for fluorescence microscopy to allow detection of NS3 and cellular proteins specified on the left of each panel. In case of Rab-7 and Rab-21, cells were transfected with expression constructs encoding GFP-tagged proteins 24 h prior to infection with Jc1. Left panels represent low magnification overviews; boxed areas are shown as enlargement in the corresponding right panel. Scale bars represent 10 µm (left panels) and 2 µm (right panels). Numbers in the right panels indicate Pearson's correlation coefficient as a marker for the degree of colocalization.
(TIFF)

Figure S3 **Impact of used EM method on morphology and size of double membrane vesicles.** (A) Huh7.5 cells grown on 6 cm-diameter dishes were infected with Jc1 (MOI = 5) and 48 h later cells were fixed, scrapped off the culture dish and sedimented by gentle centrifugation prior to embedding of the cell pellet in epon resin as described in Protocol S1 in Text S1. Owing to centrifugation cells appear much thinner than cells fixed on sapphire discs (used in most experiments) or coverslips (depicted in panel B). DMVs were detected at high abundance in the cytoplasm; average diameter was 170 nm (±46 nm; n = 30). (B) HCV-infected (MOI = 10) Huh7.5 cells grown on coverslips were subjected to chemical fixation prior to embedding in epon (Protocol S1 in Text S1). With this method the core of lipid droplets is well preserved, but DMVs and MMVs display an amorphous shape, which is at variance to their circular shape as detected after HPF-FS and chemical fixation. This is most likely due to dehydration of the cell occurring during sample preparation. DMVs detected after epon embedding displayed a diameter of ∼186 nm (±25 nm; n = 30). (C) HCV-infected Huh7.5 cells grown on sapphire discs were subjected to chemical fixation and subsequent HPF-FS as described in materials and methods. Due to the excellent preservation of the cellular membranes this was our method of choice for the EM analyses (Figures 2, 3, 6, 7 and 8).
(TIFF)

Figure S4 **Immuno-EM approaches and their impact on detection of HCV antigen and dsRNA.** (A) Jc1-infected cells (MOI = 30) were subjected to pre-embedding labeling (Protocol S2 in Text S1) by using the NS5A-specific monoclonal antibody 9E10 prior to incubation with secondary antibody conjugated with nanogold particles and subsequent signal enhancement. Although specific immuno-labeling was detected, structures were only poorly preserved and therefore the allocation of NS5A to a specific subcellular site was not possible. (B–F) Huh7.5 cells were infected with 100 TCID_50_/cell of Jc1, fixed, subjected to HPF-FS and embedded into the methacrylate resin Lowicryl HM20 (Protocol S3 in Text S1). Labeling was performed by using the dsRNA-specific antibody J2. (B) DsRNA labeling on infected cells. (C) Overview pictures of mock-infected cells to reveal unspecific labeling of the J2 antibody. (D) Amount of gold particles per µm^2^ in Jc1-infected versus mock-infected cells after immunolabeling with the dsRNA-specific antibody. (E) Relative labeling distribution obtained with the dsRNA-specific antibody J2. Two different labeling experiments were considered. Ca. 100 immuno-gold clusters were counted per grid and allocated to subcellular sites specified in the bottom. Numbers refer to the percent of total gold clusters counted per sample. ER, endoplasmic reticulum; Cyto, cytosol; Mito, mitochondria; NE, nuclear envelope; EE/LE, early/late endosomes; PM, plasma membrane; if & m, intermediate filaments and microtubule; DMVs, double membrane vesicles; LDs, lipid droplets. (F) Location of dsRNA labeling relative to DMVs. Note that ∼20% of DMVs were labeled either on their membranes or in the interior of the DMV.
(TIFF)

Figure S5 **Morphologies of the membranous web and the double membrane vesicles are independent from used cell clone, route of HCV RNA delivery, HCV genotype and MOI.** (A) Naïve high-passage Huh7 cells were infected with 100 TCID_50_/cell of Jc1 and processed after chemical fixation by HPF-FS as described in materials and methods. Note that these cells display the same kind of membrane alterations as HCV-infected Huh7.5 cells (Figure 3) demonstrating that HCV-induced membrane rearrangements are not cell clone dependent. Average diameter of DMVs (172 nm±23 nm, n = 30) was well comparable to the one observed in infected Huh7.5 cells. (B) Huh7-Lunet cells were transfected by electroporation with a subgenomic JFH1 replicon RNA and 48 h later subjected directly to HPF-FS without chemical fixation, which was necessary for biosafety reasons when using complete viral genomes. Note the high abundance of DMVs also in these native samples excluding that DMVs are an artifact caused by chemical fixation. Also note the minimal extraction of the cytosol surrounding the DMVs in comparison to chemically fixed cells. DMVs detected under these conditions had an average diameter of 162 nm (±26 nm; n = 30). (C) Huh7.5 cells containing a stably replicating subgenomic Con1 (genotype 1b) replicon, were subjected to chemical fixation prior to HPF-FS and compared to the morphology observed in Jc1-infected or JFH-1 replicon RNA-transfected cells. Note that morphologies of DMVs and MMVs are well comparable in all those cases. Thus, morphologies of DMVs and MMVs are independent from the studied HCV genotype. DMVs observed in Con1 replicon cells displayed an average diameter of 168 nm (±28 nm; n = 30). (D and E) Morphology of HCV-induced membrane rearrangements is not affected by the MOI. Huh7.5 cells were infected with only 10 TCID_50_/cell of Jc1 and processed after chemical fixation by HPF-FS 24 and 48 hpi (panels D and E, respectively). DMVs have an average diameter of 129 nm (±23 nm; n = 30).
(TIFF)

Movie S1 **Animation through a Z series of 1.184 nm thick digital slices (total thickness ∼135 nm) of a dual-axis tomogram (corresponding to** **Figure 6** **), reconstructed from a ∼250 nm thick section of a HCV-infected Huh7.5 cell, fixed 16 hpi.** Colored overlay shows a 3D surface model of virus-induced membranes. ER membranes are depicted in dark brown, inner DMV membranes in yellowish brown, outer DMV membranes in light brown, small vesicles in pink, intermediate filaments in dark blue and the Golgi stack in green. Note that most of the DMVs are connected to ER membranes via a neck-like structure.
(MP4)

Movie S2 **Animation through a Z series of 1.996 nm thick digital slices (total thickness ∼140 nm) of a dual-axis tomogram (corresponding to** **Figure 7** **), reconstructed from a ∼250 nm thick section of a HCV-infected Huh7.5 cell, fixed 36 hpi.** Colored overlay shows a 3D surface model of virus-induced membranes. ER membranes are depicted in dark brown, inner DMV, MMV and DMT membranes in yellowish brown, outer DMV, MMV and MMT membranes in light brown, small vesicles in pink, intermediate filaments in dark blue and the nucleoplasm in violet. Note that most of the DMVs are connected to ER membranes via a neck-like structure.
(MP4)

Text S1 **Supporting information.** Supplementary materials and methods (Protocols S1, S2 and S3) and supplementary Table S1.
(DOC)
