# Supplementary figures and images for: Three-Dimensional Architecture and Biogenesis of Membrane Structures Associated with Hepatitis C Virus Replication
Source: PLoS Pathog. 2012 Dec 6;8(12):e1003056. doi: 10.1371/journal.ppat.1003056 (PMC3516559; doi:10.1371/journal.ppat.1003056)

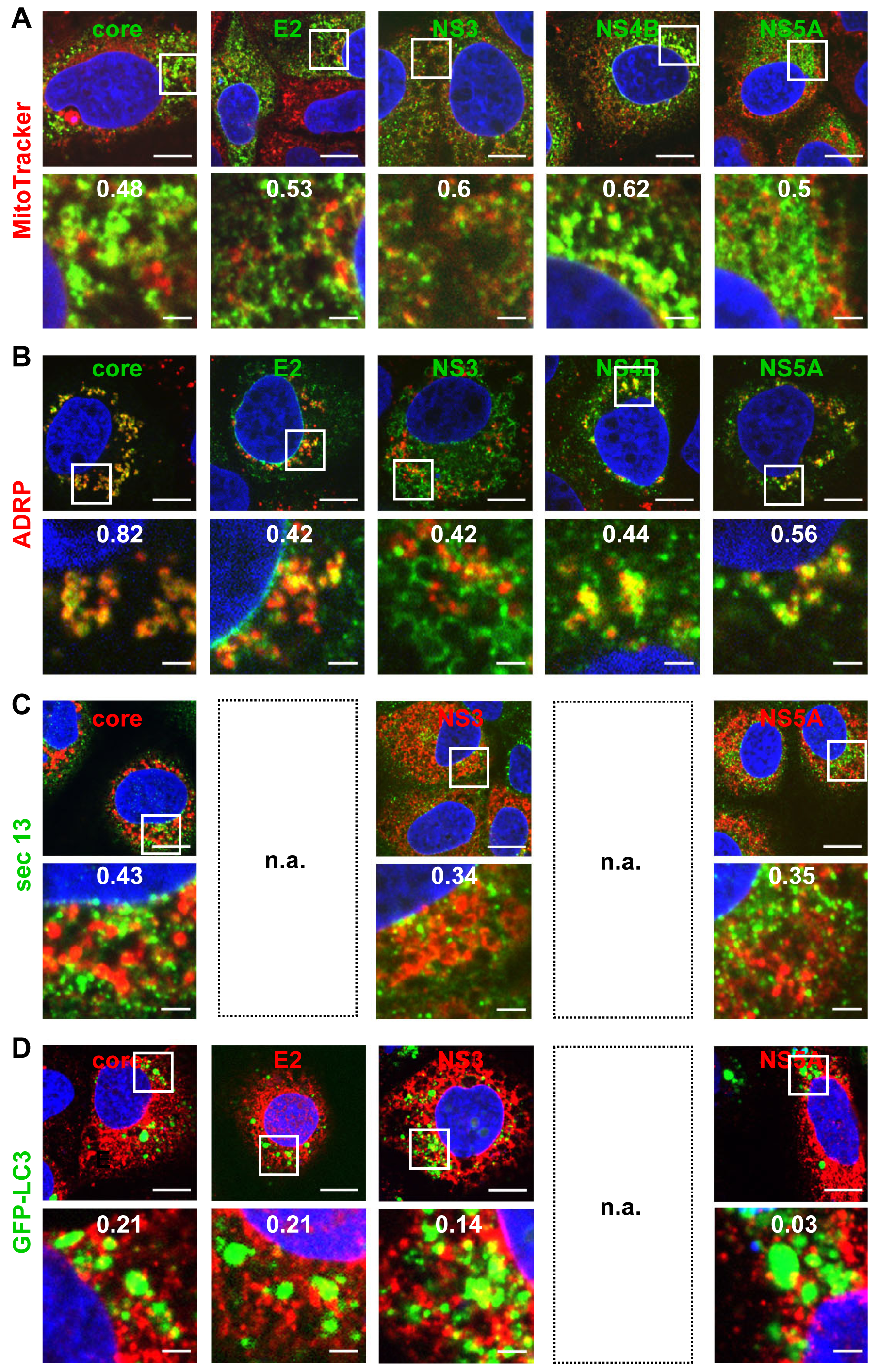

Supplement: Figure S1 — Colocalization of HCV proteins with cellular marker proteins. Huh7 cells were infected with HCV (clone Jc1) using 30 TCID50/cell and 48 h later cells were fixed and processed for fluorescence microscopy. Detected HCV proteins are specified in the top of each subpanel, cellular proteins are given in the left of each panel. Upper panels represent a low magnification overview; boxed areas are shown as enlargement in the corresponding panel below. (A)–(C) Colocalization of HCV proteins with mitochondria stained with MitoTracker, lipid droplets labeled with ADRP, or COP II vesicles labeled with sec13, respectively. (D) Cells were transfected with a GFP-LC3 expression construct and 24 h later cells were infected as described above. DNA was stained with DAPI (blue). Samples were analyzed with a Nikon TE2000-E inverted confocal microscope at 60× magnification. Scale bars represent 10 µm (top panels) and 2 µm (lower panels). Representative images are shown. The quantification of the degree of colocalization (Pearson's correlation coefficient) is given in the enlarged pictures. N.a., not applicable due to cross-reactivity of antibodies. (TIFF) [file ppat.1003056.s001.tiff]

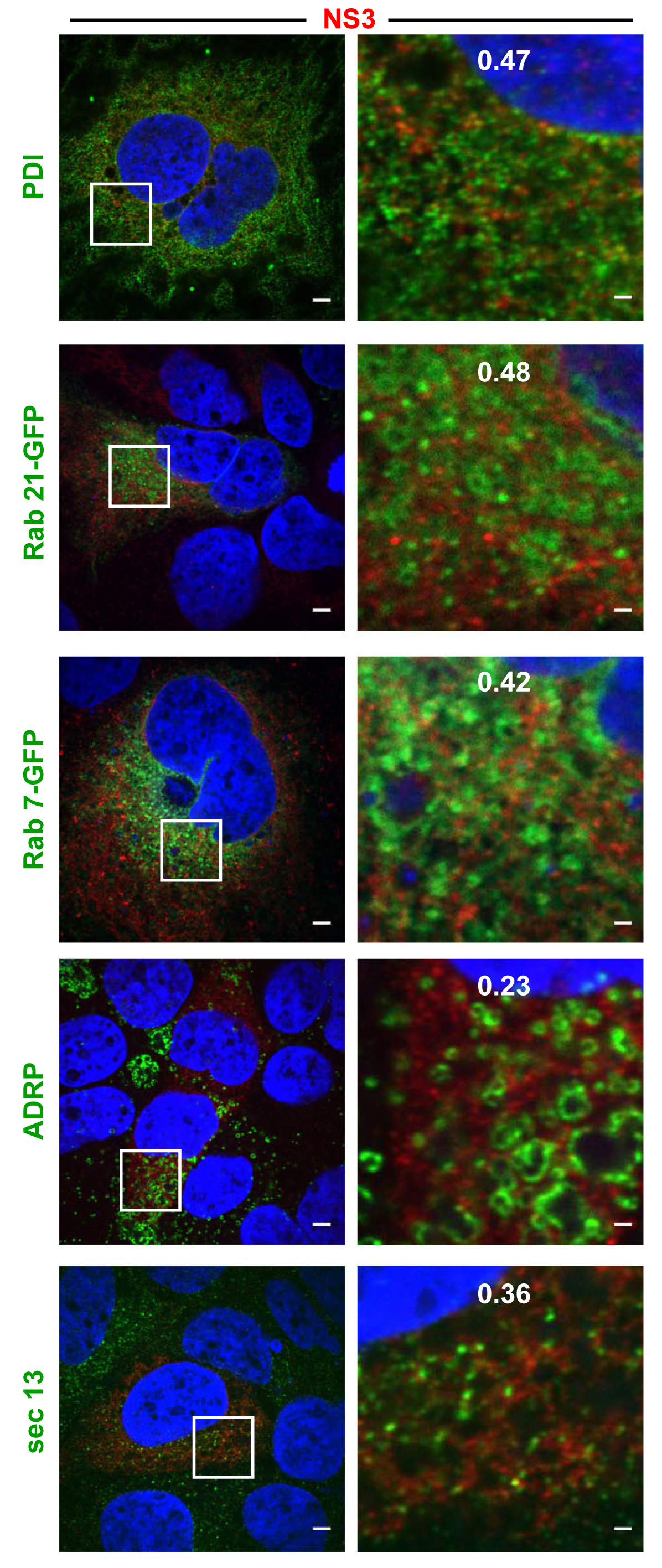

Supplement: Figure S2 — Colocalization of HCV proteins with cellular marker proteins 24 h after infection. Huh7 cells were infected with HCV (clone Jc1) using 30 TCID50/cell and 24 h later cells were fixed and processed for fluorescence microscopy to allow detection of NS3 and cellular proteins specified on the left of each panel. In case of Rab-7 and Rab-21, cells were transfected with expression constructs encoding GFP-tagged proteins 24 h prior to infection with Jc1. Left panels represent low magnification overviews; boxed areas are shown as enlargement in the corresponding right panel. Scale bars represent 10 µm (left panels) and 2 µm (right panels). Numbers in the right panels indicate Pearson's correlation coefficient as a marker for the degree of colocalization. (TIFF) [file ppat.1003056.s002.tiff]

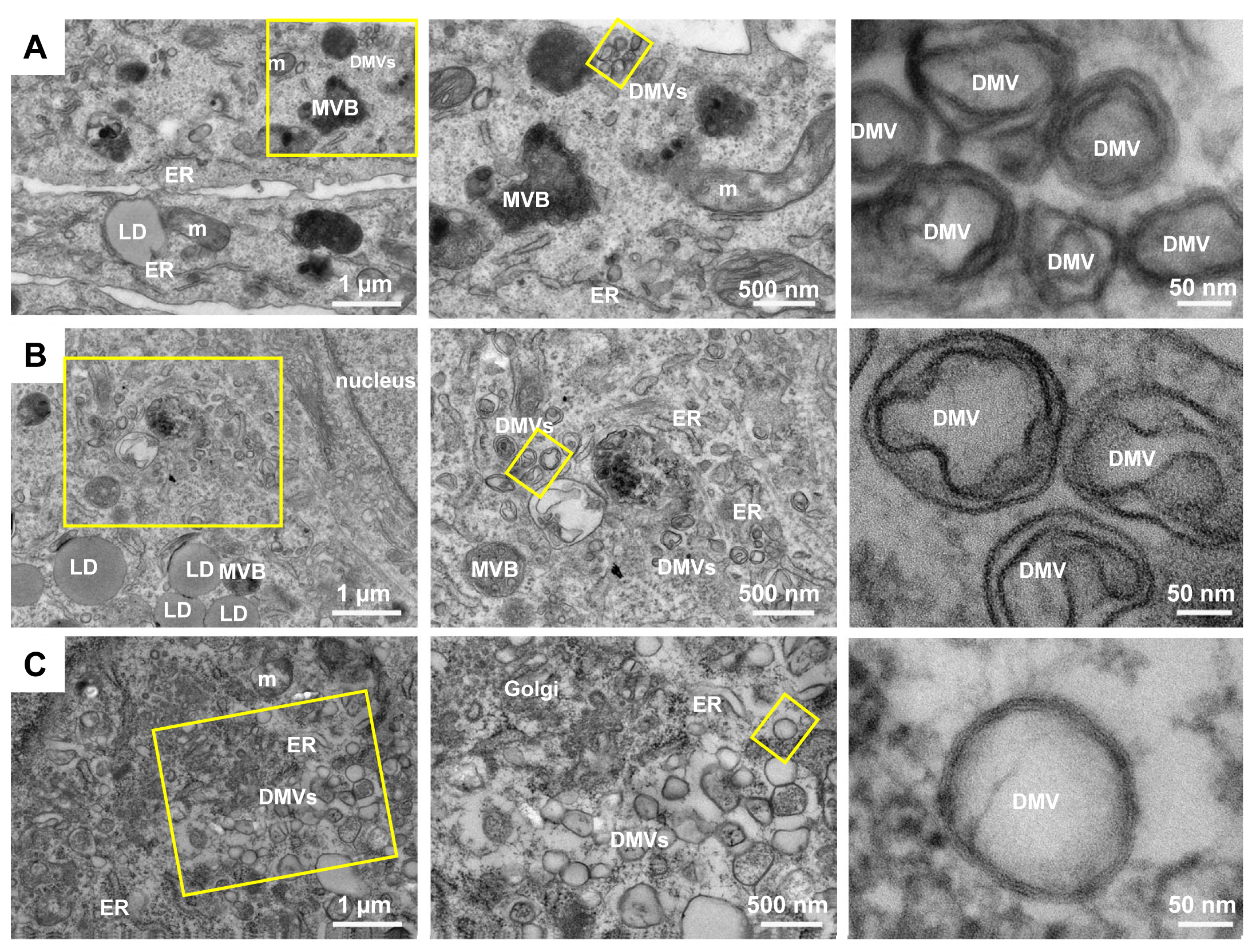

Supplement: Figure S3 — Impact of used EM method on morphology and size of double membrane vesicles. (A) Huh7.5 cells grown on 6 cm-diameter dishes were infected with Jc1 (MOI = 5) and 48 h later cells were fixed, scrapped off the culture dish and sedimented by gentle centrifugation prior to embedding of the cell pellet in epon resin as described in Protocol S1 in Text S1. Owing to centrifugation cells appear much thinner than cells fixed on sapphire discs (used in most experiments) or coverslips (depicted in panel B). DMVs were detected at high abundance in the cytoplasm; average diameter was 170 nm (±46 nm; n = 30). (B) HCV-infected (MOI = 10) Huh7.5 cells grown on coverslips were subjected to chemical fixation prior to embedding in epon (Protocol S1 in Text S1). With this method the core of lipid droplets is well preserved, but DMVs and MMVs display an amorphous shape, which is at variance to their circular shape as detected after HPF-FS and chemical fixation. This is most likely due to dehydration of the cell occurring during sample preparation. DMVs detected after epon embedding displayed a diameter of ∼186 nm (±25 nm; n = 30). (C) HCV-infected Huh7.5 cells grown on sapphire discs were subjected to chemical fixation and subsequent HPF-FS as described in materials and methods. Due to the excellent preservation of the cellular membranes this was our method of choice for the EM analyses (Figures 2, 3, 6, 7 and 8). (TIFF) [file ppat.1003056.s003.tiff]

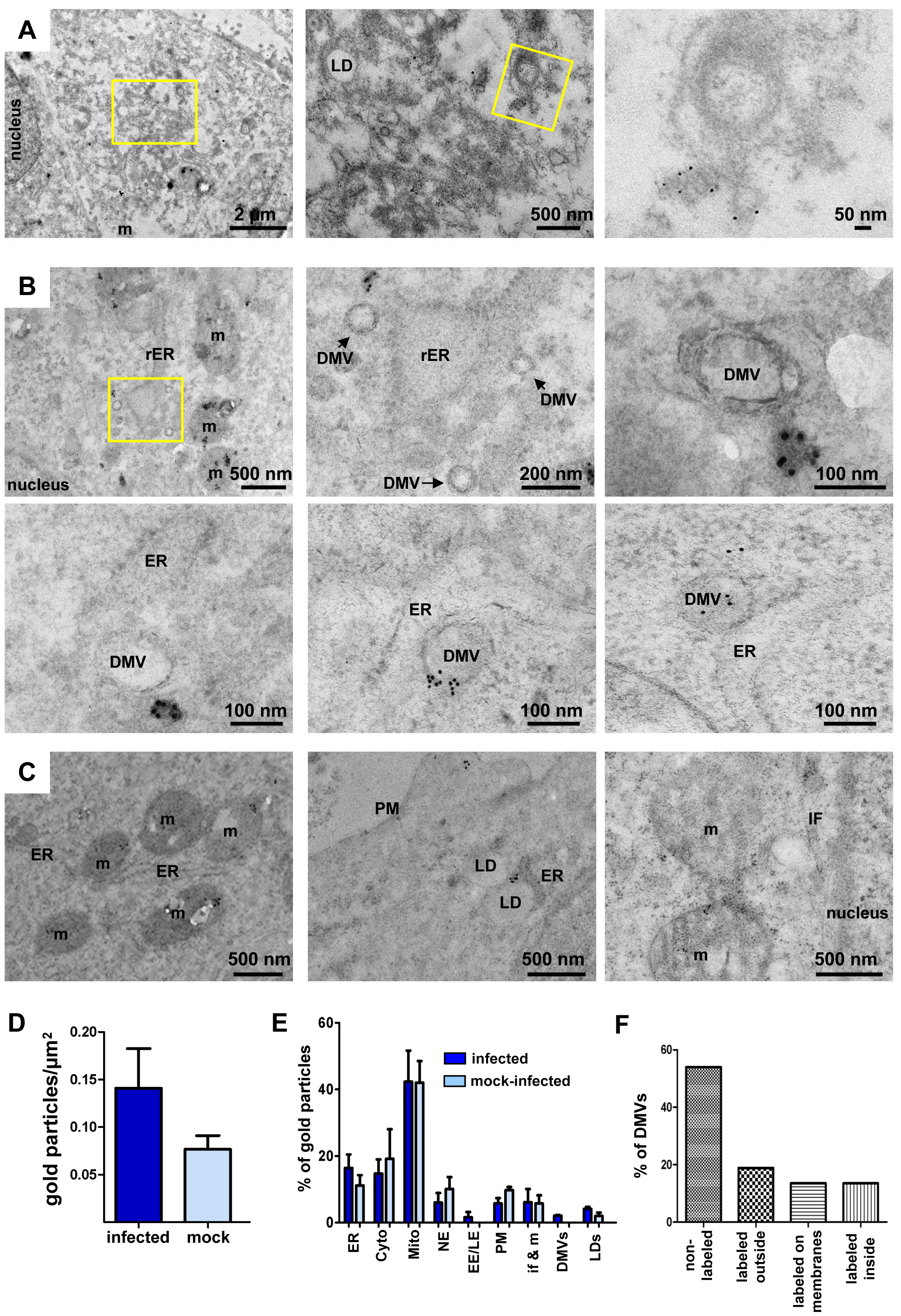

Supplement: Figure S4 — Immuno-EM approaches and their impact on detection of HCV antigen and dsRNA. (A) Jc1-infected cells (MOI = 30) were subjected to pre-embedding labeling (Protocol S2 in Text S1) by using the NS5A-specific monoclonal antibody 9E10 prior to incubation with secondary antibody conjugated with nanogold particles and subsequent signal enhancement. Although specific immuno-labeling was detected, structures were only poorly preserved and therefore the allocation of NS5A to a specific subcellular site was not possible. (B–F) Huh7.5 cells were infected with 100 TCID50/cell of Jc1, fixed, subjected to HPF-FS and embedded into the methacrylate resin Lowicryl HM20 (Protocol S3 in Text S1). Labeling was performed by using the dsRNA-specific antibody J2. (B) DsRNA labeling on infected cells. (C) Overview pictures of mock-infected cells to reveal unspecific labeling of the J2 antibody. (D) Amount of gold particles per µm2 in Jc1-infected versus mock-infected cells after immunolabeling with the dsRNA-specific antibody. (E) Relative labeling distribution obtained with the dsRNA-specific antibody J2. Two different labeling experiments were considered. Ca. 100 immuno-gold clusters were counted per grid and allocated to subcellular sites specified in the bottom. Numbers refer to the percent of total gold clusters counted per sample. ER, endoplasmic reticulum; Cyto, cytosol; Mito, mitochondria; NE, nuclear envelope; EE/LE, early/late endosomes; PM, plasma membrane; if & m, intermediate filaments and microtubule; DMVs, double membrane vesicles; LDs, lipid droplets. (F) Location of dsRNA labeling relative to DMVs. Note that ∼20% of DMVs were labeled either on their membranes or in the interior of the DMV. (TIFF) [file ppat.1003056.s004.tiff]

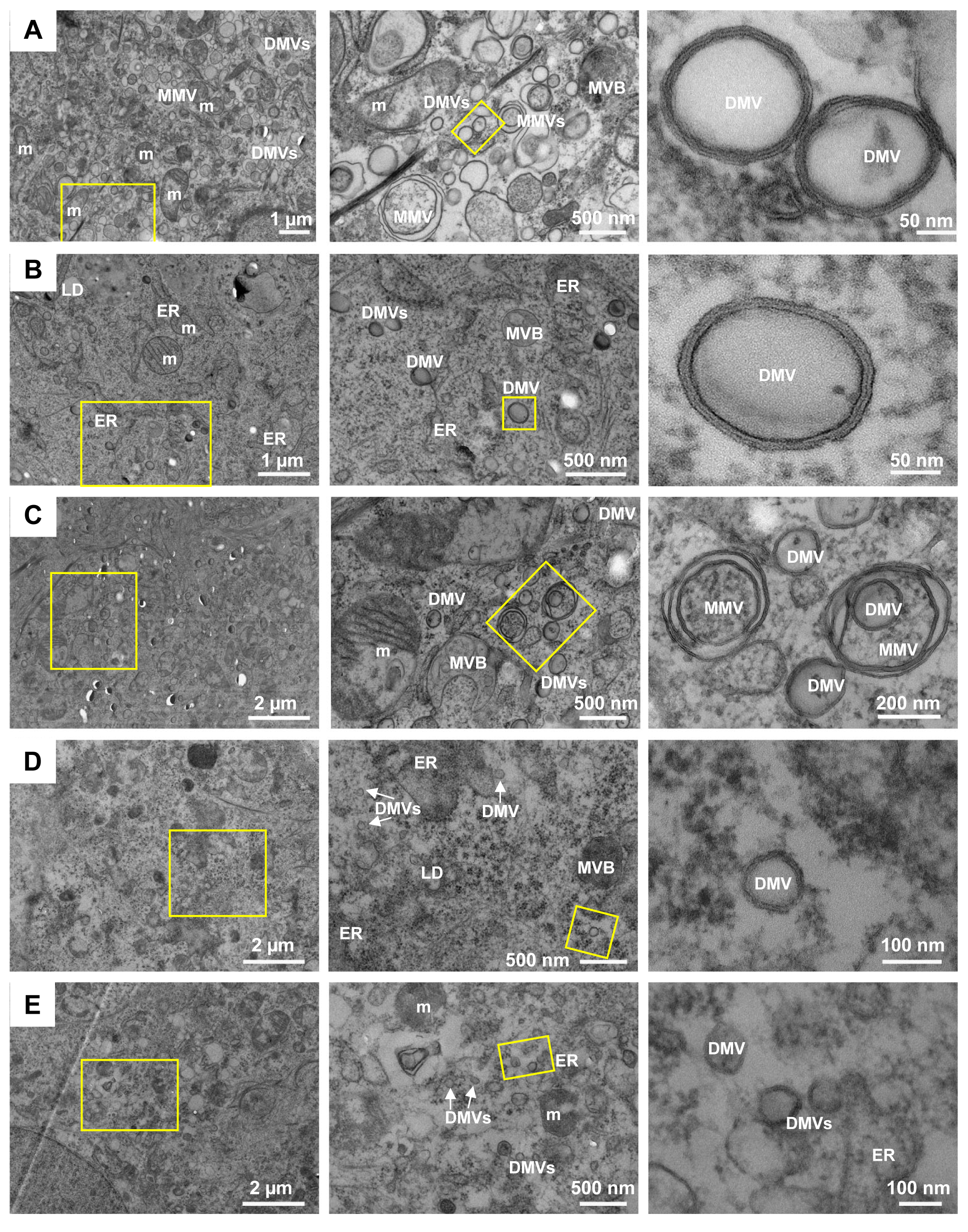

Supplement: Figure S5 — Morphologies of the membranous web and the double membrane vesicles are independent from used cell clone, route of HCV RNA delivery, HCV genotype and MOI. (A) Naïve high-passage Huh7 cells were infected with 100 TCID50/cell of Jc1 and processed after chemical fixation by HPF-FS as described in materials and methods. Note that these cells display the same kind of membrane alterations as HCV-infected Huh7.5 cells (Figure 3) demonstrating that HCV-induced membrane rearrangements are not cell clone dependent. Average diameter of DMVs (172 nm±23 nm, n = 30) was well comparable to the one observed in infected Huh7.5 cells. (B) Huh7-Lunet cells were transfected by electroporation with a subgenomic JFH1 replicon RNA and 48 h later subjected directly to HPF-FS without chemical fixation, which was necessary for biosafety reasons when using complete viral genomes. Note the high abundance of DMVs also in these native samples excluding that DMVs are an artifact caused by chemical fixation. Also note the minimal extraction of the cytosol surrounding the DMVs in comparison to chemically fixed cells. DMVs detected under these conditions had an average diameter of 162 nm (±26 nm; n = 30). (C) Huh7.5 cells containing a stably replicating subgenomic Con1 (genotype 1b) replicon, were subjected to chemical fixation prior to HPF-FS and compared to the morphology observed in Jc1-infected or JFH-1 replicon RNA-transfected cells. Note that morphologies of DMVs and MMVs are well comparable in all those cases. Thus, morphologies of DMVs and MMVs are independent from the studied HCV genotype. DMVs observed in Con1 replicon cells displayed an average diameter of 168 nm (±28 nm; n = 30). (D and E) Morphology of HCV-induced membrane rearrangements is not affected by the MOI. Huh7.5 cells were infected with only 10 TCID50/cell of Jc1 and processed after chemical fixation by HPF-FS 24 and 48 hpi (panels D and E, respectively). DMVs have an average diameter of 129 nm (±23 nm; n = 30). (TIFF) [file ppat.1003056.s005.tiff]
