# Supplementary material for: Three-Dimensional Architecture and Biogenesis of Membrane Structures Associated with Hepatitis C Virus Replication
Source: PLoS Pathog. 2012 Dec 6;8(12):e1003056. doi: 10.1371/journal.ppat.1003056 (PMC3516559; doi:10.1371/journal.ppat.1003056)
Supplement: Text S1 — Supporting information. Supplementary materials and methods (Protocols S1, S2 and S3) and supplementary Table S1. (DOC) [file ppat.1003056.s008.doc]

**Supporting information**

**Supplementary Materials and Methods**

# Protocol S1. Conventional embedding of cells for electron microscopy. Huh7.5 cells seeded onto coverslips (in case of flat embedding) or grown on 6 cm diameter dishes (in case of cell pellet embedding) were infected with 100 TCID_50_/cell of Jc1. After 48 h cells were washed 3 times with pre-warmed PBS and fixed for 30 min with 2.5% glutaraldehyde (GA) in 50 mM Na-cacodylate buffer [pH 7.4] containing 1 M KCl, 0.1 M MgCl_2_, 0.1 M CaCl_2_ and 2% sucrose. Cells were washed 5 times for 5 min each with 50 mM Na-cacodylate buffer and post-fixed on ice in the dark with 2% OsO_4_ in 50 mM Na-cacodylate buffer for 40 min. After washing the cells overnight in distilled water they were treated with 0.5% uranylacetate (UA, dissolved in water) for 30 min, rinsed thoroughly with water and dehydrated in a graded ethanol series at room temperature (40%, 50%, 60%, 70% and 80%, 5 min each; then 95% and 100%, 20 min each). Cells were immersed in 100% propylene oxide and immediately embedded in an Araldite-epon mixture (Araldite 502/Embed 812 kit, Electron Microscopy Sciences). After polymerization at 60°C for 2 days embedded cells were sectioned using a Leica Ultracut UCT ultramicrotome and a 35° diamond knife (Diatome, Biel, Switzerland). Sections with a thickness of 60 nm were collected onto 100 mesh copper grids that had been coated with Formvar (Plano, Wetzlar, Germany) and coated with carbon. Sections were counter-stained with 3% UA in 70% methanol for 5 min and 2% lead citrate in H_2_O for 2 min. Samples were analyzed by using a Biotwin CM120 Philips electron microscope (100 kV) equipped with a bottom-mounted 1K CCD camera (Keen View, SIS, Münster, Germany).

**Protocol S2. Pre-embedding immunolabeling.** Cells seeded on coverslips were infected with Jc1 and fixed with 4% PFA and 0.05% GA in 0.15 M HEPES buffer (ph 7.4) for 10 min. Subsequently cells were fixed with 4% PFA for 30 min, washed thoroughly with PBS and incubated with blocking solution (50 mM NH_4_Cl, 0.005% saponin, 0.5% BSA in PBS, pH 7.4) for 20 min. Incubation with primary antibody (diluted in blocking solution) was carried out overnight at 4°C. After several washes with PBS, samples were incubated with secondary antibody (FluoroNanogold anti-rabbit or anti-mouse Fab’-Alexa Fluor 488, Nanoprobes, NY, USA) in the dark for 2 h. After washing with PBS, gold enhancement was conducted by using the Goldenhace-EM kit (Nanoprobes) according to the instructions of the manufacturer. Cell pellets were finally embedded in epon resin as described above.

**Protocol S3. Immunolabeling of cells embedded into Methacrylate resin.** Thin sections of cells embedded into Lowicryl HM20 were incubated in blocking solution (1.5% [w/v] BSA and 0.1% [w/v] fish skin gelatin in PBS) for 20 min at room temperature. Sections were incubated with primary antibody diluted in blocking solution for 45 min at room temperature, washed with PBS and incubated for 20 min with protein A coupled to 10-nm gold particles (Cell Microscopy Center, Utrecht, The Netherlands) that had been diluted in blocking buffer. For labeling with mouse monoclonal antibodies sections were incubated with bridging polyclonal rabbit anti-mouse immunoglobulins (Dako Cytomation, Hamburg, Germany) prior to incubation with protein A gold. Cells were washed with PBS, post-fixed with GA (1% [w/v] in PBS) and washed extensively with water. Cells were stained and examined by EM as described above.

**Table S1. Primary antibodies used in this study.**

| **Specificity** | | **Name** | **Host** | **Description** | **Reference/Company** |
| --- | --- | --- | --- | --- | --- |
| **cell protein-specific antibodies** | **dsRNA** | **J2** | Mouse | MAb IgG2a | English & Scientific Consulting, Szirak, Hungary |
|  | **rough ER** | **PDI** | Mouse | Monoclonal | Iowa hybridoma Bank, Iowa, USA |
|  |  | **GRP94** | Mouse | Monoclonal IgG2a | Thermo Scientific |
|  |  | **Calnexin** | Rabbit | Polyclonal | Stressgen |
|  | **smooth ER** | **EH** | Mouse | Polyclonal | Abcam |
|  | **Reticular subdomain of rER** | **CLIMP-63** | Mouse | Monoclonal IgG2a | Alexis Biochemicals, Lausen, Switzerland |
|  | **LDs** | **ADRP** | Guinea pig | Polyclonal | Hans Heid (DKFZ, Heidelberg, Germany) |
|  | ***cis* Golgi network** | **GOS-28** | Mouse | Monoclonal IgG1 | Acris, Herford, Germany |
|  | ***trans* Golgi network** | **TGN46** | Rabbit | Polyclonal | Sigma |
|  | **autophagosomes** | **LC3** | Rabbit | Polyclonal | Cell Signalling |
|  | **COP II vesicles** | **Sec13** | Rabbit | Polyclonal | Wanjin Hong (IMB, Singapore) |
|  | **late endosome/MVB** | **Rab11** | Rabbit | Polyclonal | Cell Signaling |
|  | **lysosomes** | **Lamp-3** | Mouse | Monoclonal IgG1 | BD Pharmingen |
|  |  | **LBPA** | Mouse | Monoclonal | Jean Grünberg (University of Geneva, Switzerland) |
| **virus-specific antibodies** | **core** | **C7/50** | Mouse | Monoclonal  IgG1 | Darius Moradpour  (University of Lausanne, Lausanne, Switzerland) |
|  |  | **C830** | Rabbit | Polyclonal | [1] |
|  | **E2** | **J6E2** | Rabbit | Polyclonal | [2] |
|  | **NS3** | **2E3** | Mouse | Monoclonal | AGB (Hybridoma Bank, Iowa, USA) |
|  |  | **JFH 49** | Rabbit | Polyclonal | [2] |
|  | **NS4B** | **86** | Rabbit | Polyclonal | [3] |
|  | **NS5A** | **9E10** | Mouse | Monoclonal  IgG2a | Charles Rice  (The Rockefeller University, New York) |
|  |  | **52** | Rabbit | Polyclonal | [3] |

References

1. Koch JO, Bartenschlager R (1999) Modulation of hepatitis C virus NS5A hyperphosphorylation by nonstructural proteins NS3, NS4A, and NS4B. J Virol 73: 7138-7146.

2. Koutsoudakis G, Kaul A, Steinmann E, Kallis S, Lohmann V, et al. (2006) Characterization of the early steps of hepatitis C virus infection by using luciferase reporter viruses. J Virol 80: 5308-5320.

3. Appel N, Zayas M, Miller S, Krijnse-Locker J, Schaller T, et al. (2008) Essential role of domain III of nonstructural protein 5A for hepatitis C virus infectious particle assembly. PLoS Pathog 4: e1000035.
